# Understanding hydrothermal transformation from Mn_2_O_3_ particles to Na_0.55_Mn_2_O_4_·1.5H_2_O nanosheets, nanobelts, and single crystalline ultra-long Na_4_Mn_9_O_18_ nanowires

**DOI:** 10.1038/srep18275

**Published:** 2015-12-15

**Authors:** Yohan Park, Sung Woo Lee, Ki Hyeon Kim, Bong-Ki Min, Arpan Kumar Nayak, Debabrata Pradhan, Youngku Sohn

**Affiliations:** 1 Department of Chemistry, Yeungnam University, Gyeongsan 38541, Republic of Korea; 2 Center for Research Facilities & Department of Materials Science and Engineering, Chungnam National University, Daejeon 34134, Republic of Korea; 3 Department of Physics, Yeungnam University, Gyeongsan 38541, Republic of Korea; 4 Center for Research Facilities, Yeungnam University, Gyeongsan 38541, Republic of Korea; 5 Materials Science Centre, Indian Institute of Technology, Kharagpur 721 302, W.B., India

## Abstract

Manganese oxides are one of the most valuable materials for batteries, fuel cells and catalysis. Herein, we report the change in morphology and phase of as-synthesized Mn_2_O_3_ by inserting Na^+^ ions. In particular, Mn_2_O_3_ nanoparticles were first transformed to 2 nm thin Na_0.55_Mn_2_O_4_·1.5H_2_O nanosheets and nanobelts via hydrothermal exfoliation and Na cation intercalation, and finally to sub-mm ultra-long single crystalline Na_4_Mn_9_O_18_ nanowires. This paper reports the morphology and phase-dependent magnetic and catalytic (CO oxidation) properties of the as-synthesized nanostructured Na intercalated Mn-based materials.

Manganese (Mn) oxides are indispensable materials in many applications, particularly in batteries, fuel cells, supercapacitors, and catalysts^1^,^2^,^3^,^4^,^5^,^6^,^7^,^8^,^9^,^10^. Several attempts have been made to increase the efficiency of Mn materials (MnO_2_, Mn_2_O_3_, and Mn_3_O_4_) in the aforementioned applications. Tailoring the morphology has been a major approach and a range of morphologies have been reported, including wires/rods (1-D) and plates/sheets (2-D)^4^,^11^,^12^,^13^,^14^,^15^,^16^,^17^,^18^,^19^,^20^,^21^,^22^,^23^,^24^. Single-unit cell thick Mn_3_O_4_ sheets were synthesized by a solution method using Mn(NO_3_)_2_ and aminoethanol, which has shown a coercivity of 5.8 kOe at 5 K^21^. Tan *et al.* controlled the Mn_3_O_4_ morphology in the shape of nanowires, nanorods and nanoparticles by varying the relative amounts of cosolvents (CH_3_CN and water) using Mn(AC)_3_ precursor, and reported a large coercivity, HC = 10.7 kOe at 5 K, for the nanowires^22^. Liu *et al.* prepared single-layer MnO_2_ nanosheets via a simple one-step reaction of KMnO_4_ and sodium dodecyl sulfate (SDS), where SDS acted as the precursor of dodecanol (a reducer) and a sheet-structure agent^23^. A graphene oxide–template method was used to synthesize the MnO_2_ nanosheets with a high surface area of 157 m^2^/g and good capacitance (>1017 F/g) and rate capability (>244 F/g)^24^. For applications to batteries, the insertion/deinsertion behaviors of alkali ions (Li and Na) over Mn oxides^25^,^26^,^27^,^28^. and their synthesis/characterization have been studied^29^,^30^,^31^,^32^. Spinel LiMn_2_O_4_ has attracted the most interest as a cathode martial because of its thermal stability and high performance^2^,^7^,^14^,^25^,^33^,^34^. Zhang *et al.* prepared LiMn_2_O_4_ polyhedrons (with 200–1000 nm sizes) by a solid-state reaction using Mn_3_O_4_ nanowires and LiOH·H_2_O at 750 °C for 6 hr, and achieved a discharge capacity of 115 mAh/g^14^. As potential alternative to Li-ion batteries, Na-inserted Mn materials have attracted considerable interest owing to their lower cost (and high abundance) and similar physicochemical properties (e.g., redox potential and intercalation behavior)^29^,^30^,^31^,^35^,^36^,^37^. Recently, orthorhombic Na_4_Mn_9_O_18_ (referred to as Na_0.44_MnO_2_) has attracted a great deal of interest as a cathode material for Na-ion rechargeable batteries^32^,^38^,^39^,^40^,^41^,^42^,^43^,^44^,^45^,^46^,^47^,^48^,^49^,^50^,^51^,^52^,^53^,^54^,^55^,^56^. Several methods have been used to synthesize the material, including sol-gel/high- temperature calcinations^32^,^42^,^43^,^52^, solid-state reaction^27^,^39^, thermal-conversion of a precursor^41^,polymer-pyrolysis^45^, and hydrothermal method^52^. Hosono *et al.* used a hydrothermal method (Teflon-lined autoclave at 205 °C for 2 days) using Mn_3_O_4_ powder in a 5.0 M NaOH solution and obtained single-crystalline Na_0.44_MnO_2_ nanowires with superior capacity of 120 mAh/g and high charge-discharge cyclability^52^. In these cases, the efficiency of the material was shown to be dependent on the surface area and morphology; hence, an understanding of the change in morphology during Na (or Li and K) ion-insertion is very important. Liu *et al.* prepared Na_0.44_MnO_2_ nanorods with recipes of MnSO_4_, KMnO_4_ and NaOH solutions by a hydothermal method^56^. Le *et al.* reported a change in morphology (from nanosheets to nanowires) and crystal structure (from Mn_2_O_3_ to birnessite and Na_0.44_MnO_2_) after the hydrothermal reaction of Mn_2_O_3_ powder in a 5.0 M NaOH solution^48^. Although many studies have reported the electrochemical properties of Na-inserted MnO_x_ materials^32^,^38^,^39^,^40^,^41^,^42^,^43^,^44^,^45^,^46^,^47^,^48^,^49^,^50^,^51^,^52^,^53^,^54^,^55^,^56^, this study examined the undiscovered Na-insertion and morphological behaviors of Mn_2_O_3_ nanoparticles during a hydrothermal reaction process.

This paper reports a facile process to control the morphology and phase of alkali metal intercalated Mn oxides using a simple hydrothermal technique. Three different alkali metals (Li, Na, and K) were intercalated into the Mn_2_O_3_ powder (particles) to nanosheets, nanobelts and nanowires. In particular, quantum-thick Na_0.55_Mn_2_O_4_·1.5H_2_O nanosheets, nanobelts and single crystalline ultra-long Na_4_Mn_9_O_18_ nanowires were produced by inserting Na with different concentrations and reaction durations. The magnetic and catalytic (CO oxidation) properties of the as-synthesized Mn oxides are reported in detail. In addition to the new findings of the morphological behaviors (by Na-insertion)/detailed characterization and magnetic properties, the laser-induced Na-deinsertion behavior was also examined by Raman spectroscopy. The present study provides several new insights into the development of alkali metal ion intercalated Mn materials.

## Results and Discussion

**Figure 1:**
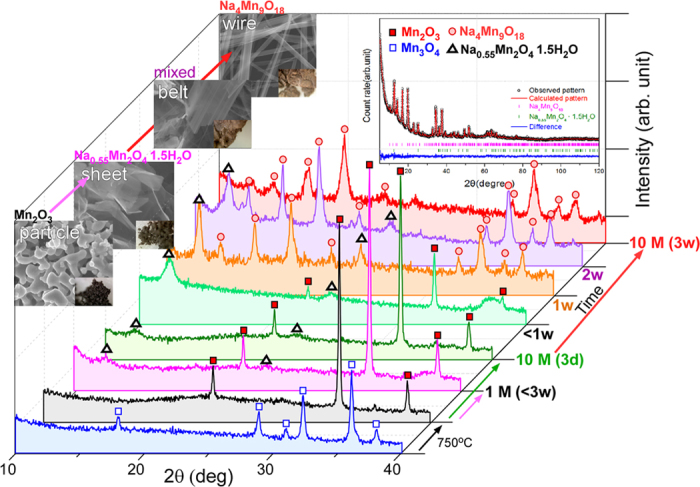
XRD patterns of the starting materials (Mn_3_O_4_ and Mn_2_O_3_) and the synthesized materials according to the reaction time in the 1.0 and 10 M NaOH solution. The insets show the corresponding SEM images (left) and Rietveld refinement powder XRD patterns of a mixed phase sample (top right). The additional Figures are provided in the Supporting Information (Figs S1, S2, and S3a, S3b) to understand the change in the crystal phase with varying reaction conditions. The reaction time was written on the right of the corresponding XRD.

Figure 1 presents powder XRD patterns and scanning electron microscopy (SEM) images of the starting materials (Mn_3_O_4_ and Mn_2_O_3_) and the synthesized Na-intercalated Mn oxides by varying the reaction conditions. The insets in the SEM images in Fig. 1 also show photographs of the powder, indicating the change in color of the sample from black (for Mn_2_O_3_) to brown (for Na_4_Mn_9_O_18_), as the hydrothermal reaction time was increased. The XRD patterns (□) of the initial starting material synthesized by a hydrothermal method at 120 °C for 12 hrs revealed tetragonal Mn_3_O_4_. Upon annealing at 750 °C for 4 hrs, the crystal structure of tetragonal Mn_3_O_4_ (■) changed to cubic phase (la-3) Mn_2_O_3_ (JCPDS 1-071-0636). A hydrothermal reaction was then performed with the Mn_2_O_3_ nanoparticles (NPs) dispersed in 1.0 and 10 M NaOH solutions at 200 °C for different durations. With increasing hydrothermal reaction time in a 10 M NaOH solution, new XRD peaks (Δ) appeared at 12.5° and 25.1° 2θ and their intensity increased. The 2θ position of these two new peaks were in good agreement with the (001) and (002) planes of monoclinic (C2/m) Na_0.55_Mn_2_O_4_·1.5H_2_O (JCPDS 43-1456). At the same time, the intensity of the XRD peaks (■) of cubic phase Mn_2_O_3_ decreased gradually. On the other hand, for the sample prepared by treating Mn_2_O_3_ NPs hydrothermally in a 1.0 M NaOH solution at 200 °C, the intensity of these new peaks (Δ) did not increase significantly, even though the reaction was performed for 3 weeks, which was attributed to the lack of Na^+^ ions. On the other hand, in the 10 M NaOH solution, these two diffraction peaks (Δ) for Na_0.55_Mn_2_O_4_·1.5H_2_O showed significant intensities upon a reaction for less than 3 days. Upon the reaction for 1 week, the XRD peaks corresponding to the cubic phase Mn_2_O_3_ were disappeared completely. Interestingly, several new diffraction peaks (ο) appeared. With further increases in the reaction time to 1~3 weeks, the two peaks (Δ) for Na_0.55_Mn_2_O_4_·1.5H_2_O at 12.5° and 25.1° 2θ decreased in intensity. After a reaction for 3 weeks, the newly appeared peaks (ο) were mainly present, which matched orthorhombic (Pbam) Na_4_Mn_9_O_18_ (JCPDS 27-0750) (Supporting Information Fig. S1 and S2)^32^,^42^,^46^. This suggests a complete change in the crystal phase of Na_0.55_Mn_2_O_4_·1.5H_2_O to Na_4_Mn_9_O_18_ with increasing hydrothermal reaction duration to 3 weeks in 10 M NaOH at 200 °C. The high purity Na_4_Mn_9_O_18_ nanowires were finally obtained after the intermediate mixture; a mixture of Na_0.55_Mn_2_O_4_•1.5H_2_O and Na_4_Mn_9_O_18_ followed by a mixture of Na_0.55_Mn_2_O_4_•1.5H_2_O and Mn_2_O_3_. High purity Na_0.55_Mn_2_O_4_•1.5H_2_O nanosheets were not observed in the hydrothermal method.

Rietveld analysis was performed for a sample with mixed crystal phases (Na_0.55_Mn_2_O_4_·1.5H_2_O:Na_4_Mn_9_O_18_ = 22.7%:77.3%). The inset in Fig. 1 shows the observed and Rietveld refinement XRD patterns (see Supporting Information, Fig. S3). The crystal structures were fully refined, and the detailed structural parameters are provided in the Supporting Information Fig. S3, Tables S1 and S2.

**Figure 2:**
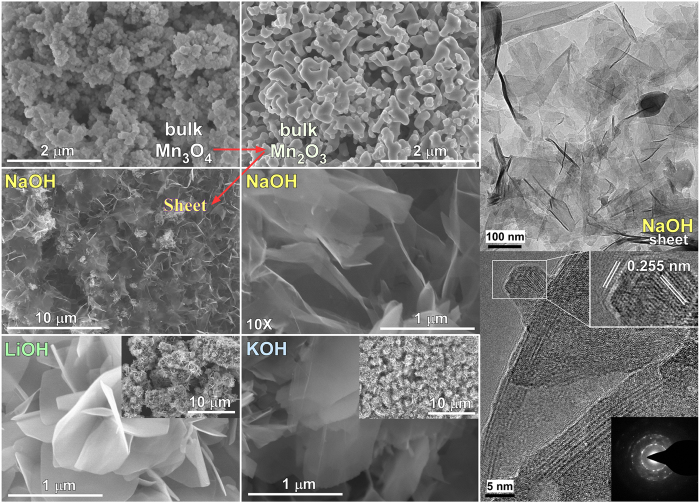
SEM images of Mn_3_O_4_, Mn_2_O_3_ and the synthesized materials in 1.0 M NaOH, LiOH, and KOH solutions. TEM and HRTEM images of the nanosheets synthesized in 1.0 M NaOH solution. The inset shows the SAED pattern of the nanosheets.

The SEM and TEM/HRTEM images of the corresponding samples were examined to further understand the recrystallization mechanism of Mn_2_O_3_ NPs in a NaOH solution under hydrothermal conditions at 200 °C for the specified duration. Figure 2 shows SEM images of the starting materials (Mn_3_O_4_ and Mn_2_O_3_) and the synthesized materials prepared by a hydrothermal method in 1.0 M NaOH, LiOH and KOH solutions for 24 hrs. The starting Mn_3_O_4_ and Mn_2_O_3_ showed particle morphologies with different sizes. On the other hand, after a hydrothermal reaction (1.0 M NaOH) at 200 °C, the surface morphology had changed entirely to ultrathin nanosheets. Under LiOH and KOH solution conditions, the surface morphologies were also changed to nanosheets, but were thicker than those prepared in the NaOH solution. Supporting Information, Fig. S4 provides additional SEM images of the nanosheets obtained by Na, Li and K intercalation. The SEM images and the XRD patterns (Fig. 1) indicate that the sheet morphology originated from the monoclinic Na_0.55_Mn_2_O_4_·1.5H_2_O phase, which was formed by the exfoliation of Mn_2_O_3_ upon Na and H_2_O concomitant intercalation. On the other hand, the presence of a Mn_2_O_3_ phase for the samples prepared in a short duration (<3 weeks in 1 M NaOH or <3 days in 10 M NaOH) was attributed to the incomplete conversion of Mn_2_O_3_ present primarily in the core part of the powder, whereas the surface consisted mainly of ultra-thin nanosheets (Fig. S5,SI). TEM, HRTEM images and electron diffraction patterns were also obtained for the ultrathin nanosheets, as shown in Fig. 2. The TEM image (top right, Fig. 2) supports the nanosheet morphology shown in the SEM images. High resolution TEM (HRTEM) (bottom right, Fig. 2) revealed the continuous lattice, indicating the crystalline nature of the nanosheets with a lattice spacing of 0.25 nm, corresponding to the (200) plane of monoclinic Na_0.55_Mn_2_O_4_·1.5H_2_O^48^. The selected area electron diffraction (SAED) patterns of the distinct spots on the rings shown as an inset of the HRTEM image further confirmed the crystalline nature of these nanosheets. More TEM and HRTEM images were provided in the Supporting Information, Fig. S5. For comparison, Ma *et al.* employed a similar hydrothermal (170 °C for 12 hrs to 1 week) method using Mn_2_O_3_ powder in a 10 M NaOH solution^57^. On the other hand, they reported Na^+^-ion free birnessite-related layered MnO_2_ nanobelts (5–15 nm width), which is inconsistent with the present study.

**Figure 3:**
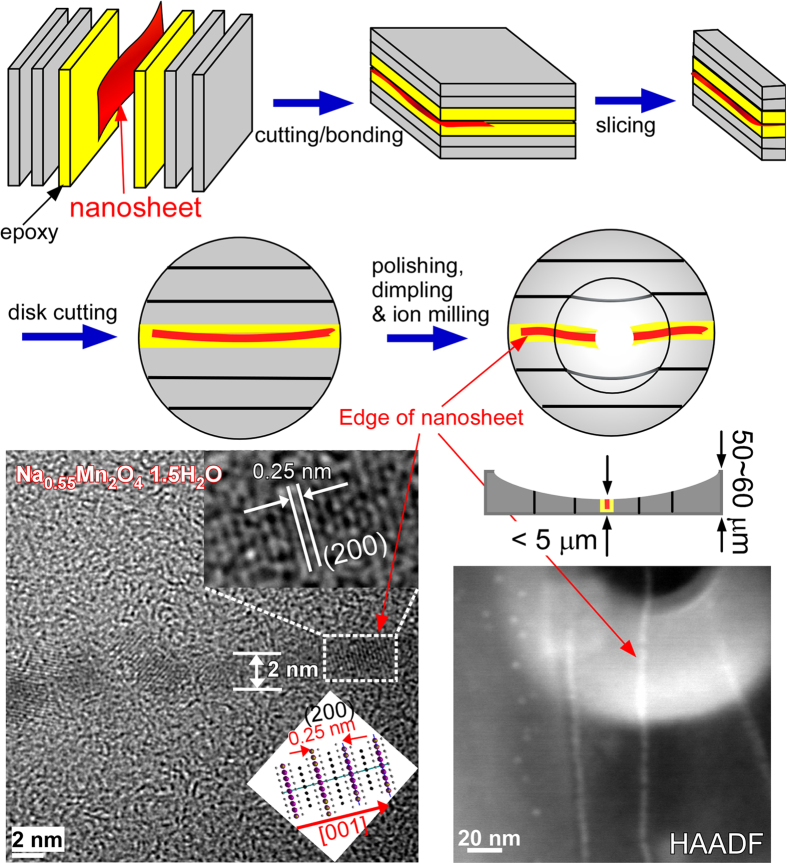
TEM sample preparation procedures (top), HRTEM image of the edge of nanosheets (bottom left). The inset shows the illustrated crystal planes. HAADF image (bottom right).

To measure the accurate thickness of the ultrathin nanosheets discussed above, a more skillful technique was employed, as described in Fig. 3. The nanosheets were first sandwiched between epoxy supported by disks, as illustrated in the Figure. Various treatment steps such as bonding, slicing, disk cutting, and ion milling, were then performed to make a suitable TEM specimen. The thickness of the TEM specimen was finally less than 5 μm. TEM, HRTEM and high-angle annular dark field (HAADF) images were taken, which clearly showed the edge of the nanosheets. Mn in the nanosheets edge was also confirmed by an EDX profile (Supporting Information, Fig. S6). The HRTEM image showed lattice fringes with neighboring distances of 0.25 nm, corresponding to the (200) plane of monoclinic Na_0.55_Mn_2_O_4_·1.5H_2_O as mentioned above. The thickness of the nanosheet edge was measured to be 2 nm, which is close to the unit cell thickness (also see Supporting Information, Fig. S7).

**Figure 4:**
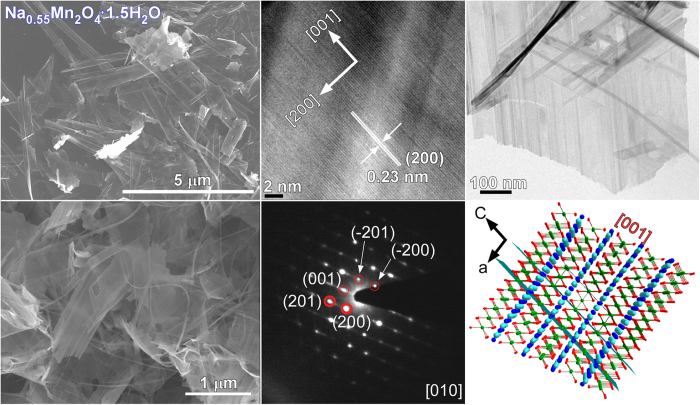
SEM (left column), low-magnification TEM, and HRTEM images of Na_0.55_Mn_2_O_4_·1.5H_2_O nanobelts. SAED and a model of the corresponding crystal planes are shown on the lower right.

**Figure 5:**
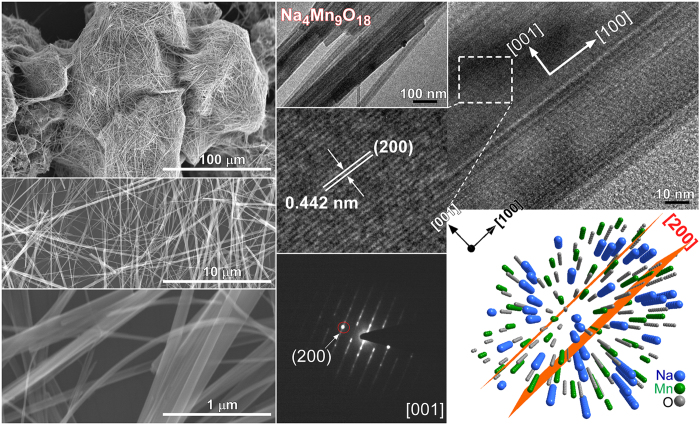
SEM (left column), low-magnification TEM and HRTEM images of Na_4_Mn_9_O_18_ nanowires. SAED and the model of the corresponding crystal planes are shown on the lower right.

**Figure 6:**
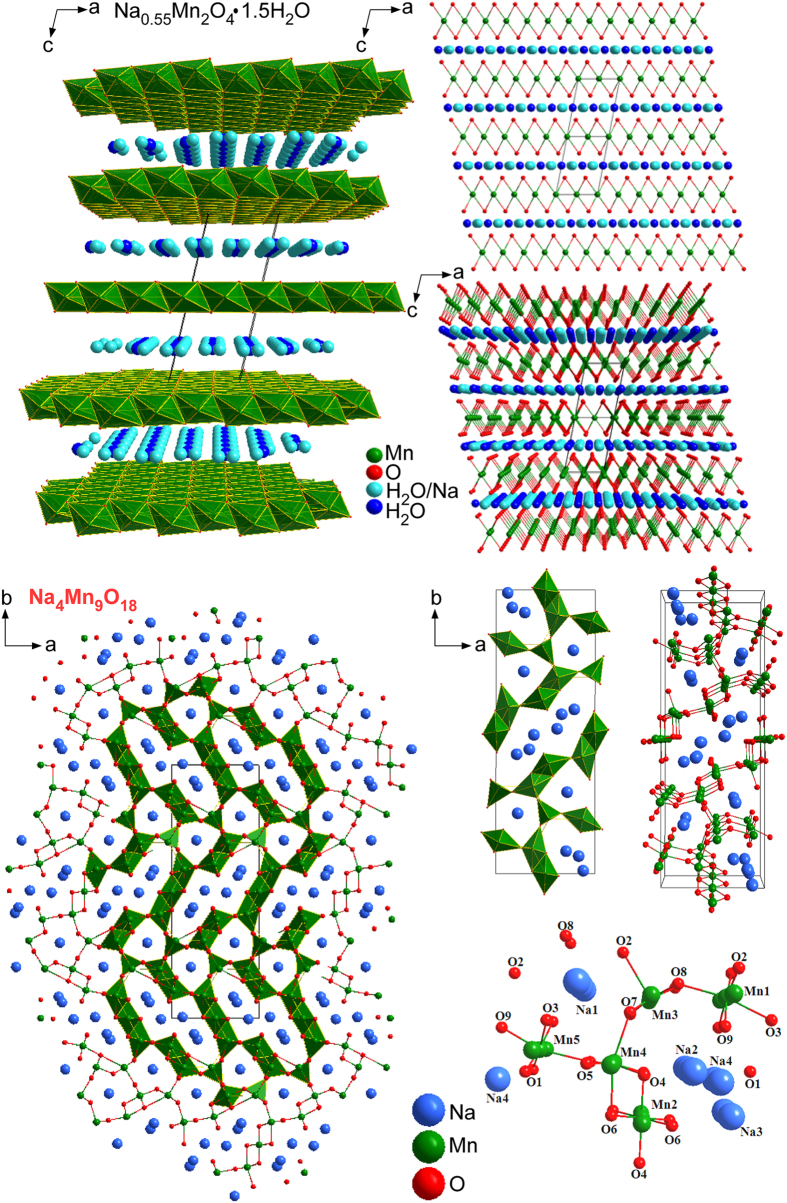
Structure projections and crystal models of Na_0.55_Mn_2_O_4_·1.5H_2_O (top) and Na_4_Mn_9_O_18_ (bottom).

Because the crystal phase of Mn_2_O_3_ was not completely changed using 1.0 M NaOH, the NaOH concentration was increased to 10.0 M and a hydrothermal reaction was performed for various reaction durations. The morphologies and microstructures of the samples obtained by the hydrothermal treatment of Mn_2_O_3_ in 10 M NaOH for 20 h at 200 °C were examined further by SEM and TEM/HRTEM, as shown in Figs 4 and 5. The Mn_2_O_3_ particles initially changed to nanosheets and nanobelts with a few nanowires (or nanothreads) for a reaction duration of less than 1 week (Supporting Information, Fig. S8), whereas the Mn_2_O_3_ nanoparticles were still present in the synthesized samples. This was supported by the corresponding XRD patterns (Fig. 1). As the reaction time increased, the nanobelts evolved slowly to ultra-long nanowires. Mixed morphologies were observed in the SEM images (Supporting Information, Fig. S9). For the corresponding XRD results (Fig. 1), the XRD patterns (∆) of Na_0.55_Mn_2_O_4_·1.5H_2_O were diminished slowly and those (ο) of Na_4_Mn_9_O_18_ were remarkable. Upon the reaction for 3 weeks, the SEM image in Fig. 5 showed mostly ultra-long (sub-mm) nanowires (also see Supporting Information, Fig. S10). The corresponding optical microscopy images showed that the black color of the Mn_2_O_3_ (with particle morphology) changed to a brown color as the crystal phase changed to Na_0.55_Mn_2_O_4_·1.5H_2_O and Na_4_Mn_9_O_18_ (Supporting Information, Fig. S11). The morphology appeared like nanofibers for the final Na-intercalated Mn product. HRTEM images of the nanobelts showed a clear lattice spacing of 0.23 nm, corresponding to the (200) plane of monoclinic Na_0.55_Mn_2_O_4_·1.5H_2_O (Fig. 4). This was also observed for the ultrathin nanosheets (Figs 2 and 3), suggesting a similar growth direction of nanosheets and nanobelts. The SAED pattern confirmed the single crystal nature of the Na_0.55_Mn_2_O_4_·1.5H_2_O nanobelts. Supporting Information, Fig. S12 shows the corresponding simulated diffraction patterns. A structure projection model in Fig. 4 displays the corresponding [001] planes of Na_0.55_Mn_2_O_4_·1.5H_2_O. Figure 5 shows representative SEM, TEM, and HRTEM images of the Na_4_Mn_9_O_18_ nanowires obtained using 10 M NaOH at 200 °C for 3 weeks. The HRTEM image shows a lattice spacing of 0.442 nm for the nanowires, which is in accordance with the (200) plane of orthorhombic Na_4_Mn_9_O_18_^32^. The spot SAED pattern confirms the single crystal structure of these nanowires. The wire grew along the [100] direction. Figure 6 shows the corresponding structure projections and crystal models of the Na-intercalated samples. In the case of the Na_0.55_Mn_2_O_4_·1.5H_2_O nanobelts, H_2_O and Na cations were concomitantly intercalated between the skeletons of Mn-O sheets. For the *ab* plane structure of the Na_4_Mn_9_O_18_ nanowires, Na was embedded in the Mn-O tunnel frame, which is consistent with the MnO_5_ square pyramids and MnO_6_ octahedra^58^. The Na cations are situated in two different sites (with a unique tunnel structure) and the *c*-axis is the charge-discharge paths of Na cation diffusion^27^,^32^,^44^. The SAED and simulated patterns of the starting material, i.e. Mn_2_O_3_, are provided in the Supporting Information, Fig. S13.

The change in crystal phase was further confirmed by FT-IR spectroscopy (Supporting Information, Fig. S14). The characteristics of the Mn-O vibrational peaks were observed between 500 and 800 cm^−1^ for all samples^13^. No OH stretching bands at approximately 3400 cm^−1^ was observed for the starting material, i.e. Mn_2_O_3_ powder. Upon the formation of Na_0.55_Mn_2_O_4_·1.5H_2_O, strong OH stretching bands were observed at 3430 and 3350 cm^−1^. On the other hand, the FTIR peaks became weaker and broader for the Na_4_Mn_9_O_18_ nanowires (Fig. 5 and Fig. S10). The much weaker broad band at 3400 cm^−1^ for Na_4_Mn_9_O_18_ was attributed to the adsorbed H_2_O (and OH) species.

**Figure 7:**
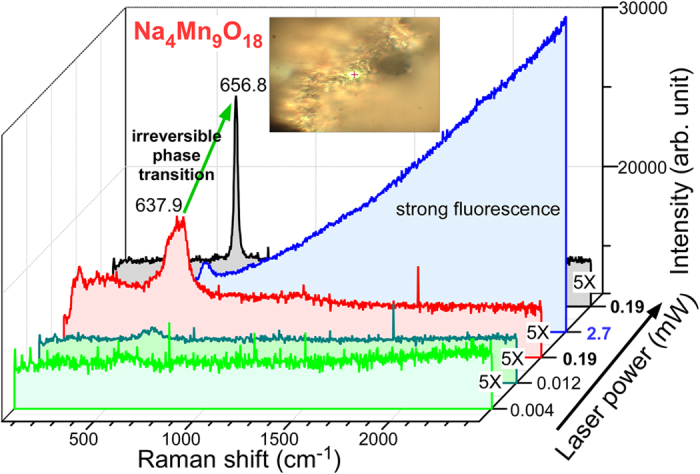
Raman spectra of the Na_4_Mn_9_O_18_ nanowires with increasing laser power. The inset shows an image of the analyzed area.

Figure 7 shows the Raman spectra of the Na_4_Mn_9_O_18_ nanowires measured with different laser powers (0.004 mW to 2.7 mW). At a low laser power (<0.012 mW), no obvious signal was observed. With increasing laser power to 0.19 mW, the Raman signals became clear at 637.9 cm^−1^ and a shoulder was observed at 561.8 cm^−1^ (see Supporting Information, Fig. S15). Upon further increases in the laser power to 2.7 mW, a strong fluorescence signal was observed (also see Supporting Information, Fig. S16) and the peak at 637.9 cm^−1^ was decreased significantly. Upon reducing the laser power to 0.19 mW, critically different Raman signals were obtained (Supporting Information, Fig. S15). This suggests that the crystal phase of Na_4_Mn_9_O_18_ had changed irreversibly to Mn_2_O_3_ by the high power laser irradiation. The laser light induces the de-insertion of Na cations in the structure, which requires further study. The newly obtained Raman spectrum shows peaks at 312.7, 374.3 and 656.8 cm^−1^, which match the bulk Mn_2_O_3_^17^. Similar Raman spectral profiles and behaviors were also observed for the Na_0.55_Mn_2_O_4_·1.5H_2_O sample (Supporting Information, Fig. S15, S16 and S17).

**Figure 8:**
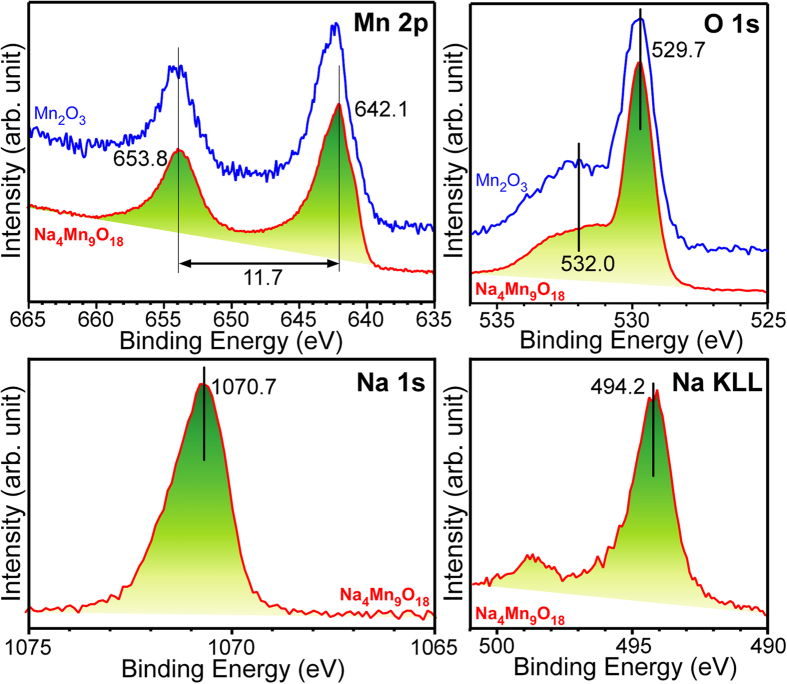
High resolution Mn 2p, O1s, N 1s, and Na KLL photoelectron spectra of the Mn_2_O_3_ particles and Na_4_Mn_9_O_18_ nanowires.

X-ray photoelectron spectroscopy (XPS) was used to examine the chemical states of Na_4_Mn_9_O_18_ nanowires and compared with those of the starting material, *i.e.*, hydrothermally synthesized Mn_2_O_3_ powders, as displayed in Fig. 8. A typical survey XPS scan of Mn_2_O_3_ showed Mn, O and impurity carbon signals, whereas that of Na_4_Mn_9_O_18_ showed additional Na as well as Mn, O and C (Supporting Information, Fig. S18). The distinct peaks at ~653.8 and ~642.1 eV (Fig. 8, top left) were assigned to the Mn 2p_1/2_ and Mn 2p_3/2_ XPS peaks, respectively, with a spin-orbit energy splitting of 11.7 eV^49^. The Mn 2p XPS peaks for Na_4_Mn_9_O_18_ were shifted slightly to a lower binding energy, confirming the Na insertion and reduction of the oxidation state of Mn^59^,^60^. The O 1s XPS spectra showed two broad peaks at 532.0 and 529.7 eV (Fig. 8, top right) due to the absorbed surface oxygen (e.g., OH, H_2_O, and O_2_) species and lattice oxygen atoms of the Mn oxides, respectively^13^. The Na 1s XPS and Na KLL Auger peaks for Na_4_Mn_9_O_18_ (Fig. 8, bottom panel) were observed at 1070.7 and 494.2 eV, respectively^49^.

**Figure 9:**
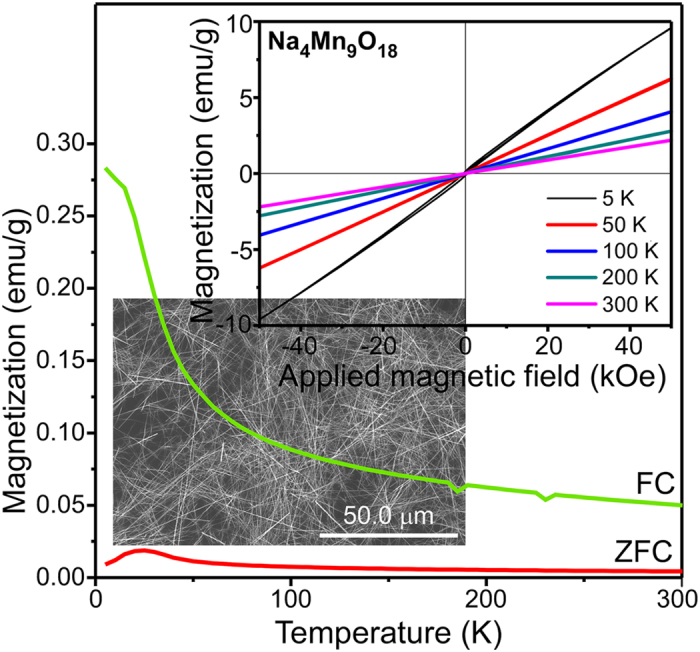
Mass-normalized FC and ZFC curves of Na_4_Mn_9_O_18_ nanowires from 5 to 300 K in H = 100 Oe. The inset show the magnetization (M−H) curves measured at various temperatures.

The magnetic properties of the Na_4_Mn_9_O_18_ nanowires were examined by SQUID. Figure 9 presents zero-field-cooling (ZFC) and field-cooling (FC) magnetization curves measured at an applied field of H = 100 Oe (0.1 kOe) over the temperature range of 5−300 K. The top inset in Fig. 9 shows the magnetization (M−H) curves measured at various temperatures from 5 K to 300 K and magnetic fields from −50 to 50 kOe. An ideal linear plot (with no hysteresis loop) of magnetization was obtained with an applied magnetic field at temperatures between 300 K and 50 K, indicating the paramagnetic and antiferromagnetic properties of the Na_4_Mn_9_O_18_ nanowires. The M−H curves showed no saturation magnetism in the external fields up to 50 kOe. A magnetization of 2.19 emu g^−1^ was measured at 50 kOe and 300 K. The mass magnetic susceptibility of the nanowires at 300 K was 4.39 × 10^−5^ emu·g^−1^·Oe^−1^. This increased with decreasing temperature and was determined to be 5.58 × 10^−5^ emu·g^−1^·Oe^−1^ at 50 K. Interestingly, a magnetic hysteresis loop was clearly observed at 5 K (Supporting Information, Fig. S19), suggesting typical ferromagnetic behavior. On the other hand, the M−H curve showed no saturation, indicating antiferromagnetic property. The residual magnetism (or remanence) and coercive force were measured to be 0.136 emu·g^−1^ and 475 Oe, respectively. A coercivity of 10.7 kOe at 5 K was reported for the Mn_3_O_4_ nanowires^22^. For single unit cell thickness Mn_3_O_4_ nanosheets, Huang *et al.* observed paramagnetic and ferromagnetic (with a coercivity of 5.8 kOe) behaviors at room temperature and 5K, respectively^21^. The FC magnetization curve increased with decreasing temperature. On the other hand, the ZFC curve was increased slowly with decreasing temperature to 25 K, and decreased below that temperature. The ZFC curve showed a maximum at 25 K. This suggests a clear transition from paramagnetic to ferromagnetic at a temperature below 25 K. The FC and ZFC curves showed no overlap at all temperatures up to 300 K.

The surface resistance of Na_4_Mn_9_O_8_ nanowires was measured as a function of temperature (Supporting Information, Fig. S20). The resistance of 12.5 MΩ at room temperature decreased linearly to 1.0 MΩ with increasing temperature to 200 °C. For the Mn_3_O_4_ (in Fig. 2) and Mn_2_O_3_ powder samples, the surface resistance could not be measured because of the high resistance.

The CO oxidation activities (Supporting Information, Fig. S21) of Mn_3_O_4_ (in Fig. 2), Mn_2_O_3_ (in Fig. 2), and Na_0.55_Mn_2_O_4_·1.5H_2_O nanosheets (or Mn_2_O_3_@Na_0.55_Mn_2_O_4_·1.5H_2_O core-shell structures; sample prepared with NaOH solution in Fig. 2) was tested for catalytic applications, such as CO oxidation using low cost materials^13^. In the first CO oxidation runs, the CO oxidation onsets were observed in the order of Mn_2_O_3_ (200 °C) < Na_0.55_Mn_2_O_4_·1.5H_2_O (250 °C) < Mn_3_O_4_ (280 °C). The T_10%_ (the temperature at 10% CO conversion) for Mn_2_O_3_, Mn_3_O_4_ and Na_0.55_Mn_2_O_4_·1.5H_2_O was observed at 240 °C, 280 °C and 320 °C, respectively. In the second runs, the order was the same as the onset temperatures of 180 °C (Mn_2_O_3_), 260 °C (Na_0.55_Mn_2_O_4_·1.5H_2_O) and 300 °C (Mn_3_O_4_). The T_10%_ for Mn_2_O_3_, Mn_3_O_4_ and Na_0.55_Mn_2_O_4_·1.5H_2_O was observed at 230 °C, 320 °C and 365 °C, respectively. Only the Mn_2_O_3_ nanoparticles showed an increase in CO oxidation activity in the second run. The Na-insertion into Mn_2_O_3_ (forming Na_0.55_Mn_2_O_4_·1.5H_2_O nanosheets on the surface) showed no synergistic effect for CO oxidation. Ji *et al.* prepared α- Mn_2_O_3_ nanowires (by a molten salt method), Mn_2_O_3_ nanoparticles and mixed Mn_2_O_3_/Na_2_Mn_8_O_16_ (a ratio of 9/1) samples, and tested their CO oxidation activities^13^. They reported that α- Mn_2_O_3_ nanowires (T_10%_ ≈ 180 °C) showed much catalytic activity than the others (T_10%_ ≈ 220 °C) and Na_2_Mn_8_O_16_ did not relate to their high catalytic activity. Their conclusions are in good agreement with the present study.

## Conclusion

Na-ion intercalation into Mn_2_O_3_ was initially transformed into ultra-thin monoclinic Na_0.55_Mn_2_O_4_·1.5H_2_O nanosheets and nanobelts. The nanobelts were then evolved to single crystalline ultra-long orthorhombic Na_4_Mn_9_O_18_ nanowires with a (Na-ion mobile) tunnel structure. This synthesis process was extended further to other alkali metals (Li and K) using a simple hydrothermal method in a Mn_2_O_3_–dispersed alkali hydroxide (LiOH, NaOH and KOH) solution. SEM and TEM confirm the transformation of the morphology. XRD and HRTEM were used to examine the crystal phase change and microstructure. Detailed crystal structural parameters were obtained by Rietveld refinement analysis. XPS confirmed the presence of inserted Na cation. Moreover, high power laser irradiation readily induces the irreversible Na-deinsertion behavior from Na_4_Mn_9_O_18_ to Mn_2_O_3_, as confirmed by Raman spectroscopy. The Na_4_Mn_9_O_8_ nanowires exhibited ferromagnetic behavior at temperatures below 25 K and paramagnetic behavior at above that temperature. The surface resistance of Na_4_Mn_9_O_8_ nanowires was 12.5 MΩ at room temperature and decreased linearly to 1.0 MΩ with increasing temperature to 200 °C. The CO oxidation activity (T_10%_ = 230 °C) of the Mn_2_O_3_ nanoparticles was substantially decreased after Na-intercalation. The very detailed transformation mechanism and the new fundamental characterization provide new insights into the development of alkali metal cation intercalated Mn oxides.

## Methods

### Material synthesis

Mn_3_O_4_ was synthesized by a hydrothermal method, as described below. Briefly, 10 mL of 0.1 M Mn(II) nitrate tetrahydrate (Sigma-Aldrich. >97.0%) was mixed with 10 mL of deionized water (18.2 MΩ cm resistivity) in a Teflon jar (120 mL capacity), and 1.0 mL of an ammonia solution was then added to obtain the precipitates. The reaction jar was capped tightly and placed in an oven (120 °C) for 12 hours, after which the oven was cooled naturally to room temperature. The brown precipitate was collected after washing with deionized water followed by ethanol, and then dried in an air convection oven (80 °C). Bulk Mn_2_O_3_ was obtained by the post-annealing of Mn_3_O_4_ at 750 °C for 4 hrs. To synthesize the Na(or Li and K)-intercalated Mn materials, the Mn_2_O_3_ (~25 mg) was dispersed in a 20.0 mL 1.0 M (or 10 M) NaOH (or LiOH and KOH) solution. The solution in a Teflon-lined stainless autoclave was placed at 200 °C for a reaction time, which was varied from 12 hrs to 3 weeks. After a specified time (12 hrs, 1 day, 3 days, 1, 2 and 3 weeks were selected to show in the present article), the oven was stopped and cooled naturally to room temperature and the powder product was collected by centrifuging. The powder was finally washed and dried for further characterization. Although the slow reaction process took time and patience (and somewhat industrially impractical) we employed the slow process to disclose new findings and to carefully examine change in morphology which has never been reported for Mn oxide material.

### Material characterization

The surface morphology of the synthesized powder samples was examined by field emission scanning electron microscopy (FE-SEM, Hitachi SE-4800). High resolution transmission electron microscopy (HRTEM) and the electron diffraction patterns were obtained using a FEI Tecnai G2 F20 at an operating voltage of 200 kV. The powder X-ray diffraction (XRD) patterns were obtained using a PANalytical X’Pert Pro MPD diffractometer operated at 40 kV and 30 mA using Cu Kα radiation. The Rietveld refinement was performed using the TOPAS software program (ver. 4.2, Bruker 2005). Further details are described elsewhere^61^. The Fourier-transform infrared (FT-IR) spectroscopy was performed using a Thermo Scientific Nicolet iS10 spectrometer in ATR (attenuated total reflectance) mode. The X-ray photoelectron spectra were obtained using a Thermoscientific K-alpha X-ray photoelectron spectrometer with a monochromated Al Kα X-ray source, a pass energy of 20.0 eV, and an analyzed spot size of 400 μm. Confocal Raman microscopy (PRISM, NOST Co., South Korea) was conducted to take the Raman spectra for the powder samples at a laser wavelength of 532 nm and a 100 ×, 0.9NA microscope objective. The laser intensity was varied from 0.004 mW to 2.7 mW. All the Raman spectra were referenced to the Raman spectrum of cyclohexane. The magnetic properties of the Na_4_Mn_9_O_18_ nanowires were examined using a MPM5-XL-7 superconducting quantum interference device (SQUID) magnetometer (Quantum Design, Inc.) at various temperatures.

### CO oxidation and surface resistance tests

The CO oxidation experiments were performed on a continuous flow quartz U-tube reactor with a 10 mg sample. A mixed gas (1% CO and 2.5% O_2_ in N_2_ balance) was introduced into the reactor at a flow rate of 40 mL/min. The temperature heating rate was fixed to 20 °C/min. The reaction gas products were analyzed using a SRS RGA200 quadrupole mass spectrometer. The surface resistance of the pelletized sample was measured using a home-built four-probe resistance measurement instrument.

## Additional Information

**How to cite this article**: Park, Y. *et al.* Understanding hydrothermal transformation from Mn_2_O_3_ particles to Na_0.55_Mn_2_O_4_·1.5H_2_O nanosheets, nanobelts, and single crystalline ultra-long Na_4_Mn_9_O_18_ nanowires. *Sci. Rep.* **5**, 18275; doi: 10.1038/srep18275 (2015).

## Supplementary Material

Supplementary Information
