# Supplementary material for: Understanding hydrothermal transformation from Mn2O3 particles to Na0.55Mn2O4·1.5H2O nanosheets, nanobelts, and single crystalline ultra-long Na4Mn9O18 nanowires
Source: Sci Rep. 2015 Dec 15;5:18275. doi: 10.1038/srep18275 (PMC4678907; doi:10.1038/srep18275)
Supplement: Supplementary Information [file srep18275-s1.pdf]

Supporting Information

Understanding hydrothermal transformation from Mn_2O_3 particles to $Na_{0.55}Mn_2O_4 \cdot 1.5H_2O$ nanosheets, nanobelts, and single crystalline ultra-long $Na_4Mn_9O_{18}$ nanowires

Yohan Park,[1,+] Sung Woo Lee,[2,+] Ki-Hyeon Kim,[3] Bong-Ki Min,[4] Arpan Kumar Nayak,[5] Debabrata Pradhan,[5,*] and Youngku Sohn[1,*]

[1]*Department of Chemistry, Yeungnam University, Gyeongsan, Gyeongbuk 38541, Republic of Korea*

[2]*Center for Research Facilities & Department of Materials Science and Engineering Chungnam National University, Daejeon 34134, Republic of Korea*

[3]*Department of Physics, Yeungnam University, Gyeongsan 38541, Republic of Korea*

[4]*Center for Research Facilities, Yeungnam University, Gyeongsan, Gyeongbuk 38541, Republic of Korea*

[5]*Materials Science Centre, Indian Institute of Technology, Kharagpur 721 302, W.B., India*

* Corresponding author e-mails:youngkusohn@ynu.ac.kr; deb@matsc.iitkgp.ernet.in

[+] There authors equally contributed to this work.

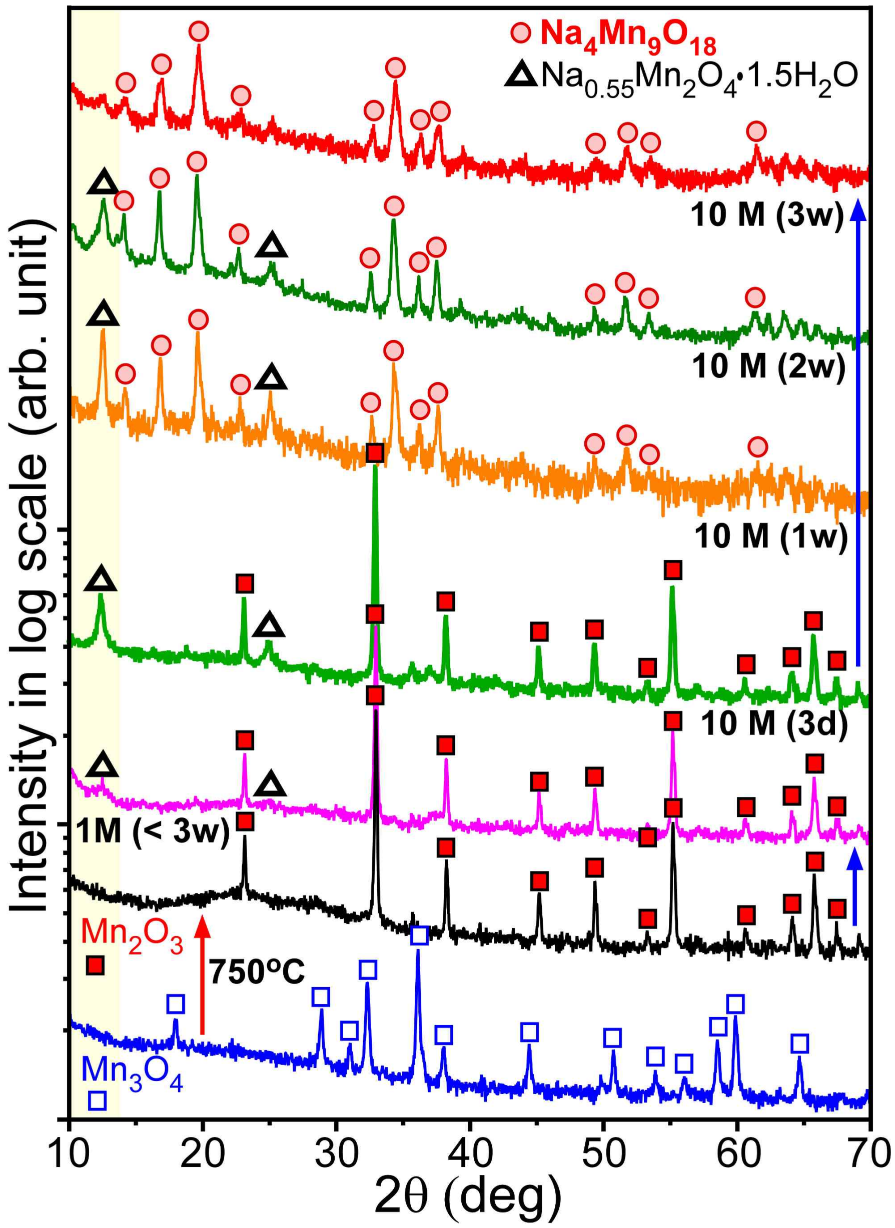

Fig. S1. XRD patterns of the starting materials and the synthesized materials with reaction time in 1.0 and 10 M NaOH solution conditions.

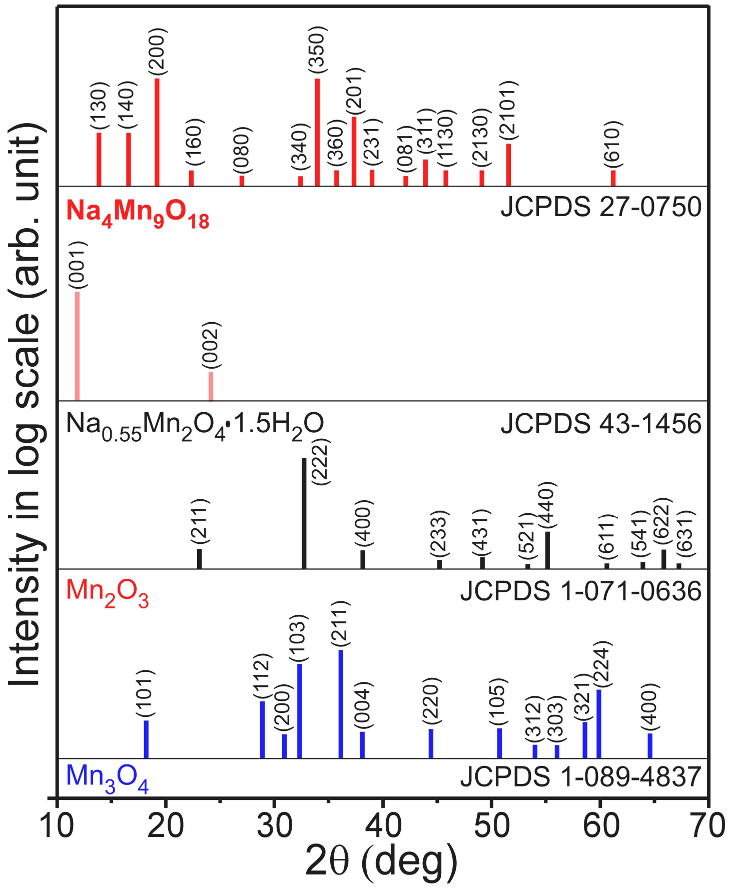

Fig. S2. Standard XRD patterns of Mn_3O_4, Mn_2O_3, $Na_{0.55}Mn_2O_4{\cdot}1.5H_2O$, and $Na_4Mn_9O_{18}$.

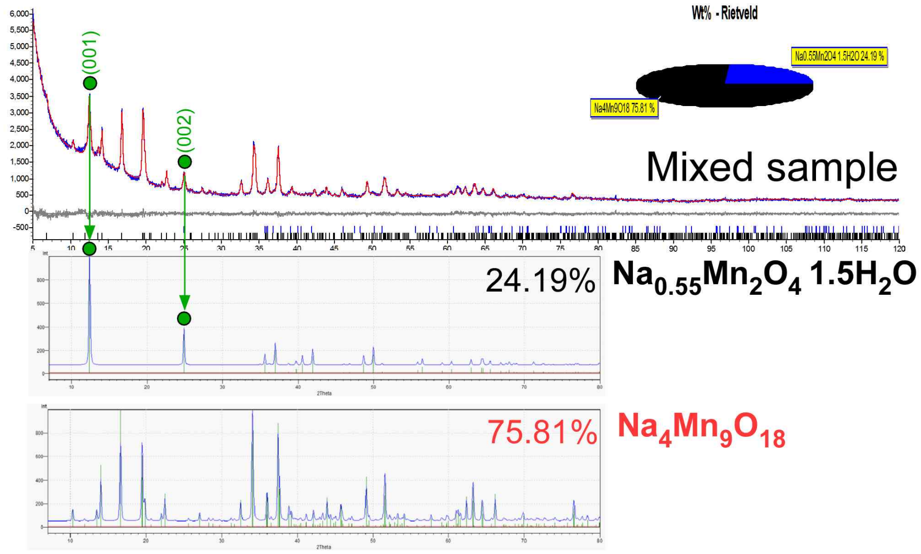

Fig. S3a. Mixed (two crystal phases) XRD patterns and resolved two pure XRD patterns. The mixed sample showed a composition ratio of 24.19%:75.81% of $Na_{0.55}Mn_2O_4{\cdot}1.5H_2O{:}Na_4Mn_9O_{18}$.

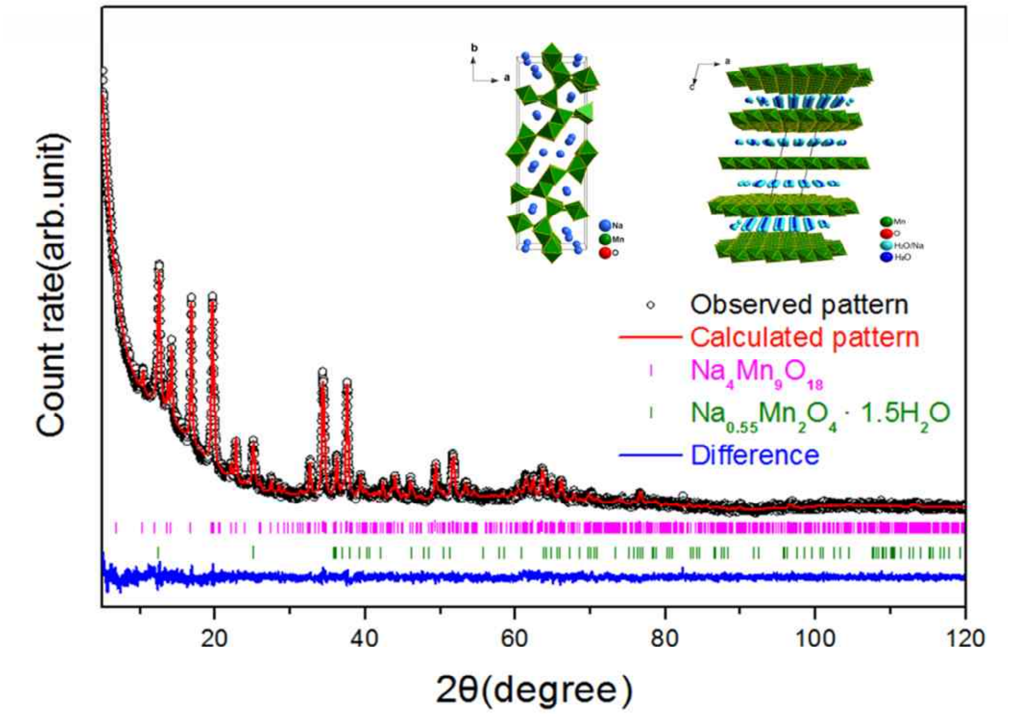

Fig. S3b. Observed (O) and Rietveld refinement X-ray powder diffraction patterns of a mixed phase sample (**Fig. S3a**). The difference plot (blue –) is shown at the bottom. Tick marks (green and pink | for $Na_{0.55}Mn_2O_4{\cdot}1.5H_2O$ and $Na_4Mn_9O_{18}$, respectively) above the difference plot indicate the Bragg reflection positions, identified by Rietveld analysis. The red solid line is the calculated pattern.

Table S1. Refined crystal structural parameters of $Na_4Mn_9O_{18}$ and $Na_{0.55}Mn_2O_4{\cdot}H_2O$ obtained using the Rietveld refinement analysis of X-ray powder diffraction data acquired at room temperature. The Oc and *B*iso represent the occupation and isotropic thermal parameters, respectively. The numbers in parentheses are the estimated standard deviations of the last significant digit. The atom crystallographic position, R-factors and a-lattice parameters are summarized in Table. The isotropic temperature factors (B) were obtained.

Reliability factors and goodness of fit

Rexp = 3.75%, Rwp = 4.35, Rp = 3.30, GOF = 1.16

Refined crystal structural parameters of $Na_4Mn_9O_{18}$

Atom	site	*x*	*y*	*z*	*Oc*	*B*iso
Na1	4g	0.2200(29)	0.2125(13)	0.00000	0.630(21)	1
Na2	4h	0.7099(39)	0.0791(15)	0.50000	0.481(25)	1
Na3	4g	0.1175(11)	-0.0105(78)	0.00000	0.570(21)	1
Na4	4g	0.8369(88)	0.0620(34)	0.00000	0.228(26)	1
Mn1	4h	0.87688(99)	0.19177(34)	0.50000	1	1
Mn2	2c	0.50000	0.00000	0.00000	1	1
Mn3	4g	0.53908(73)	0.19259(32)	0.00000	1	1
Mn4	4h	0.37009(92)	0.08883(32)	0.50000	1	1
Mn5	4g	0.0296(10)	0.11004(29)	0.00000	1	1
O1	4h	0.9820(31)	0.07606(81)	0.50000	1	1
O2	4g	0.9142(37)	0.23325(83)	0.00000	1	1
O3	4h	0.0766(33)	0.15628(87)	0.50000	1	1
O4	4g	0.5092(30)	0.07300(98)	0.00000	1	1
O5	4g	0.2625(28)	0.0902(11)	0.00000	1	1
O6	4h	0.3649(32)	0.0080(11)	0.50000	1	1
O7	4h	0.4475(32)	0.16866(97)	0.50000	1	1
O8	4h	0.6612(28)	0.2128(11)	0.50000	1	1
O9	4g	0.8549(33)	0.1456(10)	0.00000	1	1

Space group: *Pbam* (No.55)

a = 9.09544(63) b = 26.0536(21) c = 2.82987(14) Å, R_{Bragg} = 1.020

Phase composition (wt%) via Rietveld refinement : 75.81%

Refined crystal structural parameters of $Na_{0.55}Mn_2O_4 \cdot H_2O$

Atom	site	*x*	*y*	*z*	*O*	*B*iso
Mn	2a	0.00000	0.00000	0.00000	1	18.4(39)
O	4i	0.366(14)	0.00000	0.2893(92)	1	1
Na	4i	0.727(13)	0.00000	0.50000	0.70(16)	15.7(18)
Wat.1	4i	0.727(13)	0.00000	0.50000	0.30(16)	15.7(18)
Wat.2	2c	0.00000	0.00000	0.50000	1	15.8(24)

Space group: *C12/m1* (No.12)

a = 5.0621(72) b = 2.9029(29) c = 7.2498(10) Å, β= 100.85(10), R_{Bragg} = 0.259

Phase composition(wt%) via Rietveld refinement : 24.19%

Table S2. Selected bond distances (Å)

$Na_4Mn_9O_{18}$		$Na_{0.55}Mn_2O_4 \cdot H_2O$	
Mn-O	d(Å)	Mn-O	d(Å)
Mn(1) - O(2)	1.813(3)(x2)	Mn(1)-O1	2.524(x6)
O(3)	2.038(2)(x1)		2.739(x8)
O(8)	2.036(2)(x1)	<Mean value>	2.63
O(9)	1.868(3)(x2)		
<Mean value>	1.94		
Mn(2)-O(4)	1.904(4)(x2)		
O(6)	1.886(2)(x4)		
<Mean value>	1.89		
Mn(3)-O(2)	2.242(2) (x1)		
O(7)	1.757(3) (x4)		
O(8)	1.874(2) (x2)		
<Mean value>	1.96		

Mn(4)- O(4)	1.941(4) (x2)
O(5)	1.721(3) (x2)
O(6)	2.107(3) (x1)
O(7)	2.195(3) (x1)
<Mean value>	1.99
Mn(5)-O(1)	1.724(3) (x2)
O(3)	1.907(3) (x2)
O(5)	2.181(3) (x1)
O(7)	1.840(3) (x1)
<Mean value>	1.91

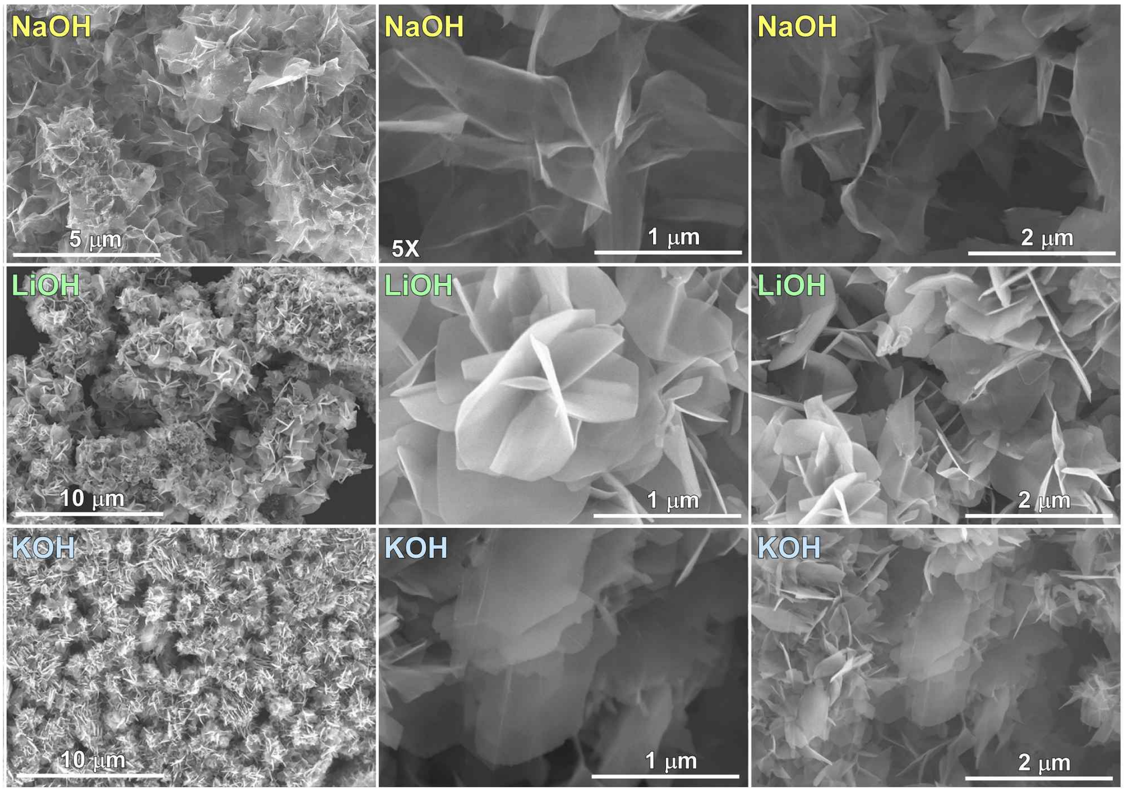

Fig. S4. SEM images of the materials synthesized in 1.0 M NaOH, LiOH, and KOH solutions for 24 hours.

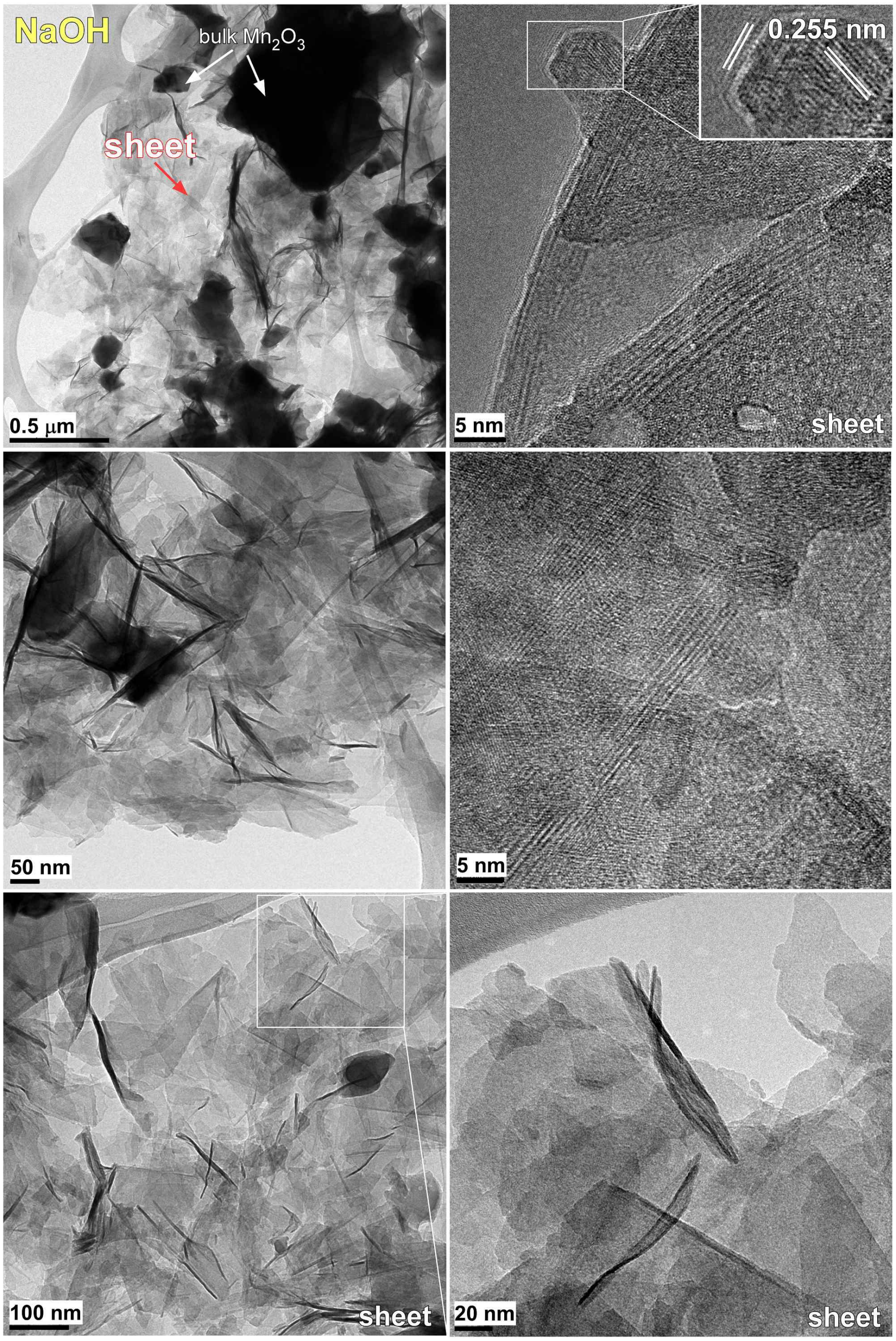

Fig. S5. TEM and HRTEM images of the ultrathin nanosheets prepared in 0.1 M NaOH solution. Bulk Mn_2O_3 nanoparticles are also present as well as the nanosheets.

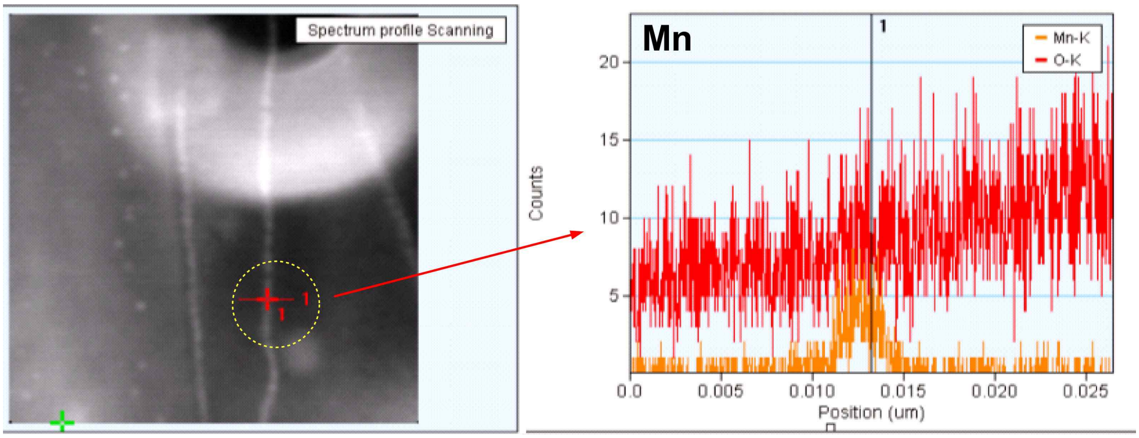

Fig. S6. HAADF image (left) of the sample and EDX profiles (right) of a nanowire edge.

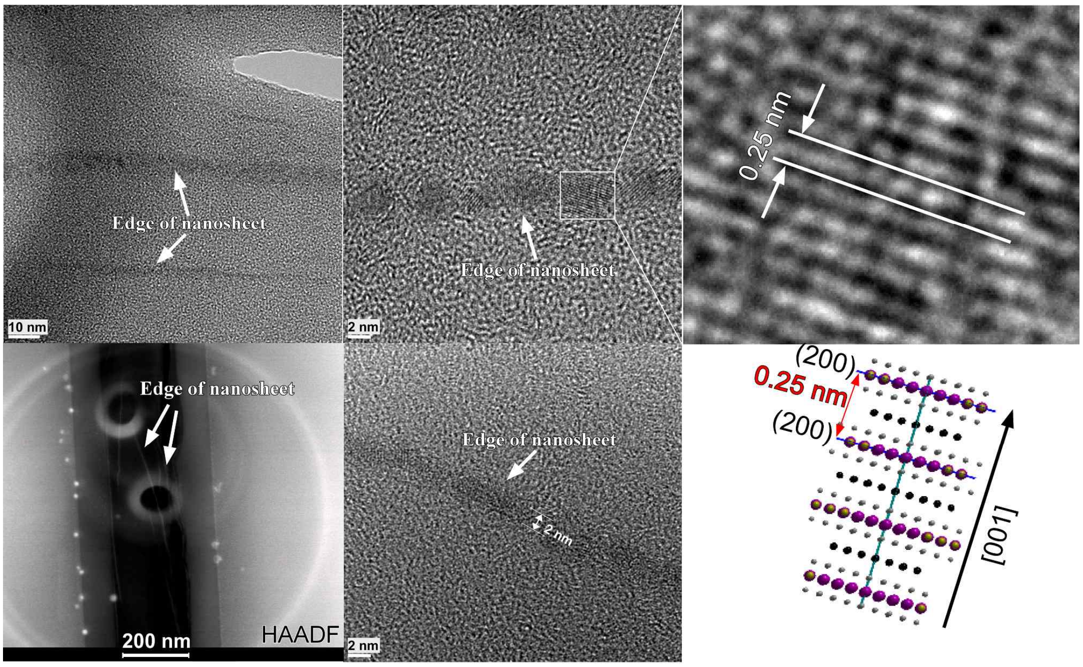

Fig. S7. TEM and HRTEM image of the edges of nanosheets, HAADF image (bottom left), Illustrated (200) crystal plane (bottom right) showing [001] direction.

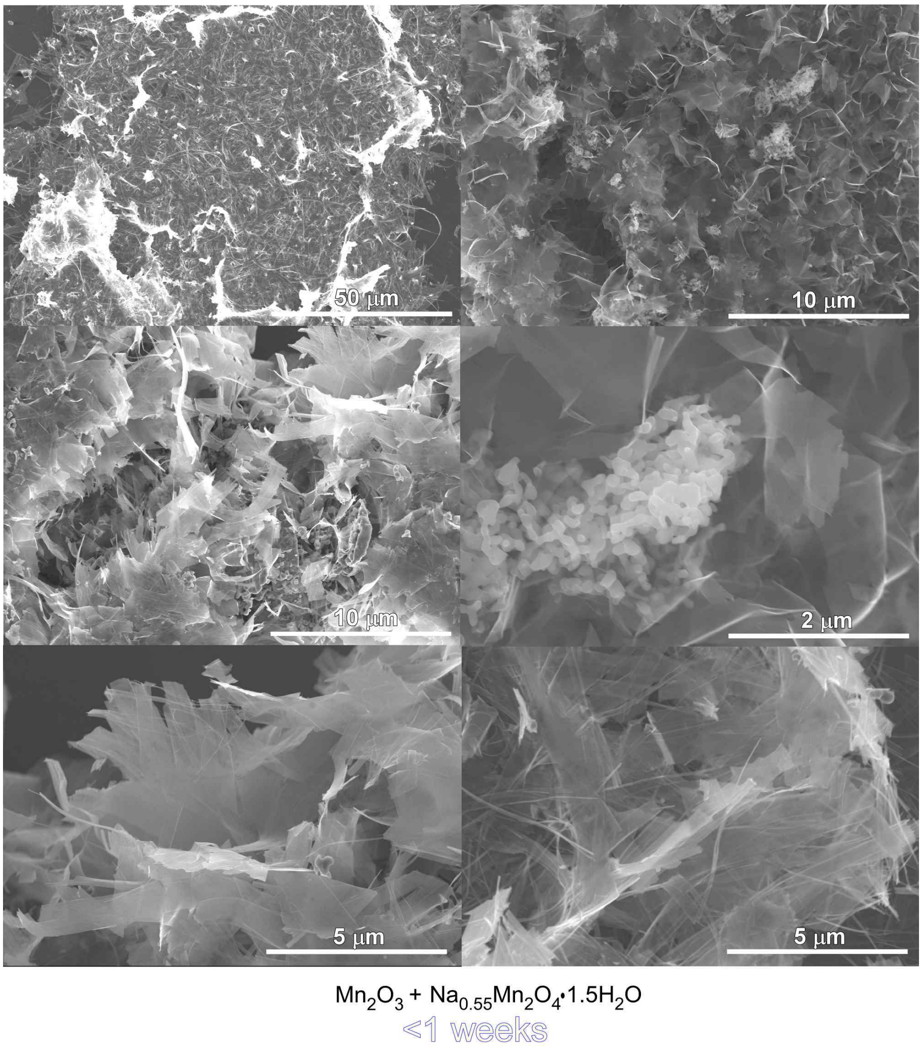

Fig. S8. SEM images of mixed samples upon reactions in 10 M NaOH solution for < 1 week.

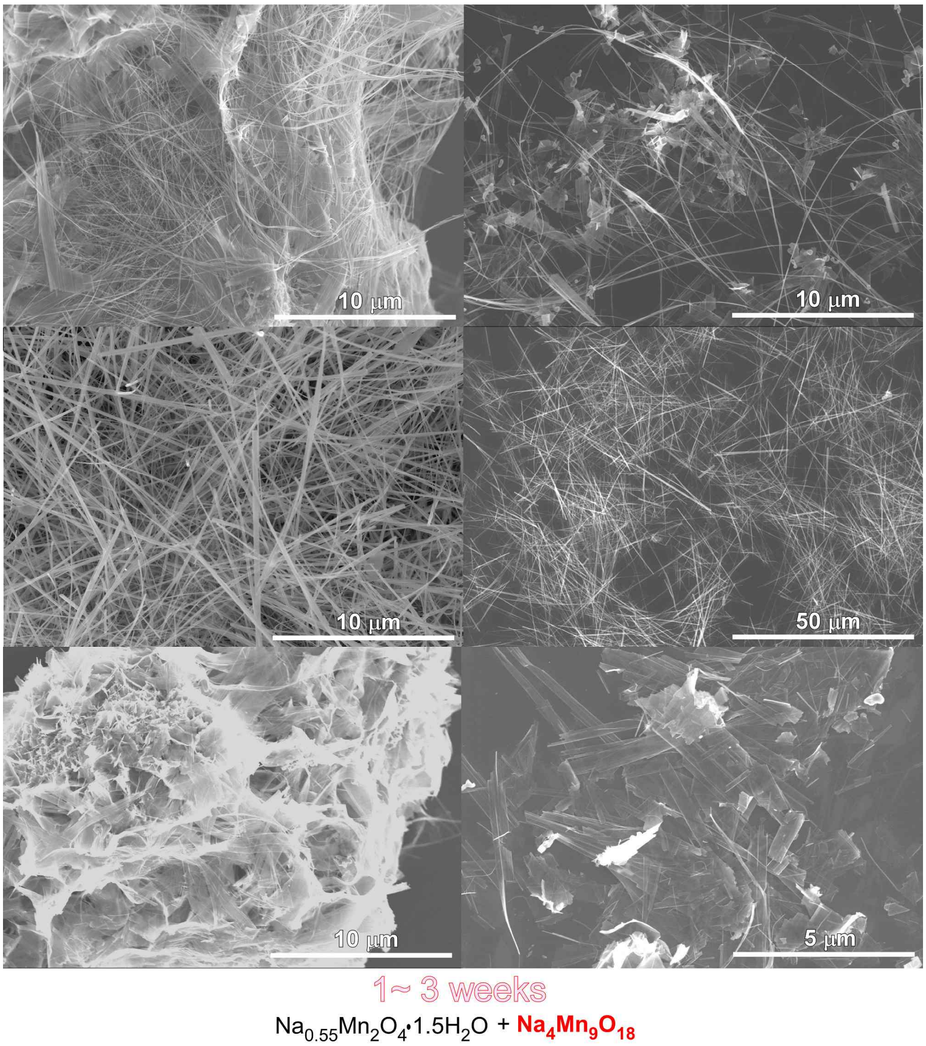

Fig. S9. SEM images of mixed samples upon reactions in 10 M NaOH solution for 1 ~ 3 weeks.

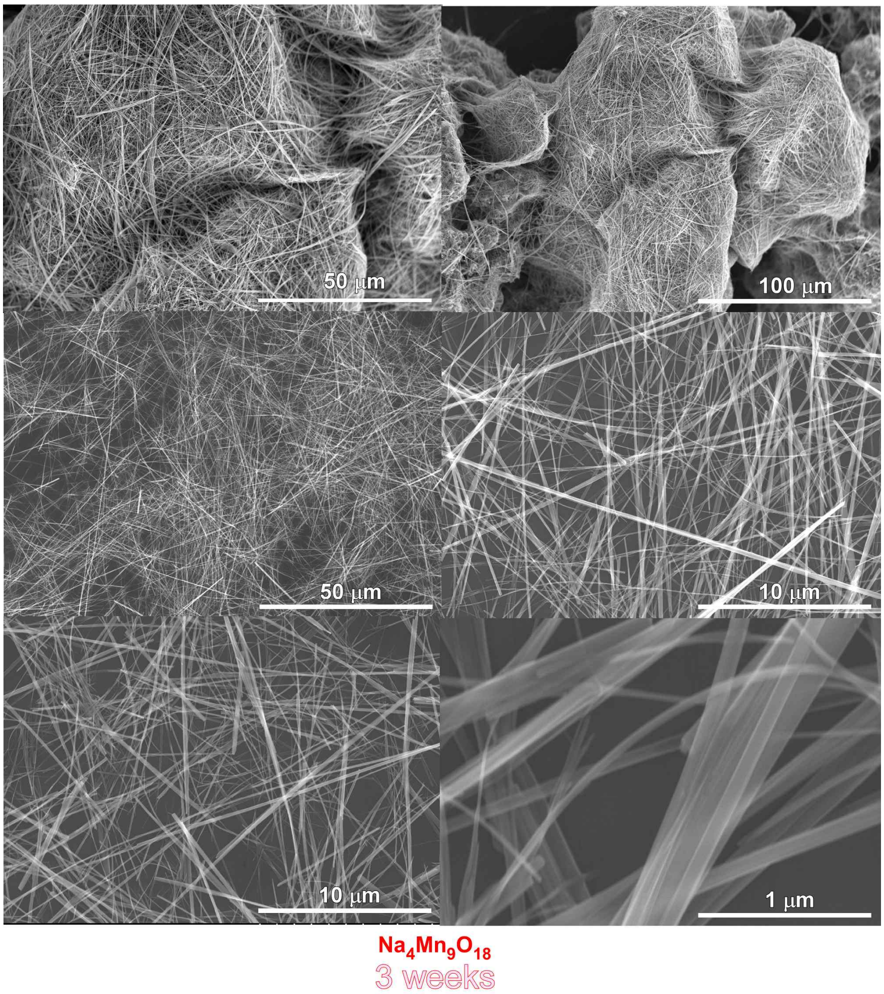

Fig. S10. SEM images of $Na_4Mn_9O_{18}$ sample upon reaction in 10 M NaOH solution for ~ 3 weeks.

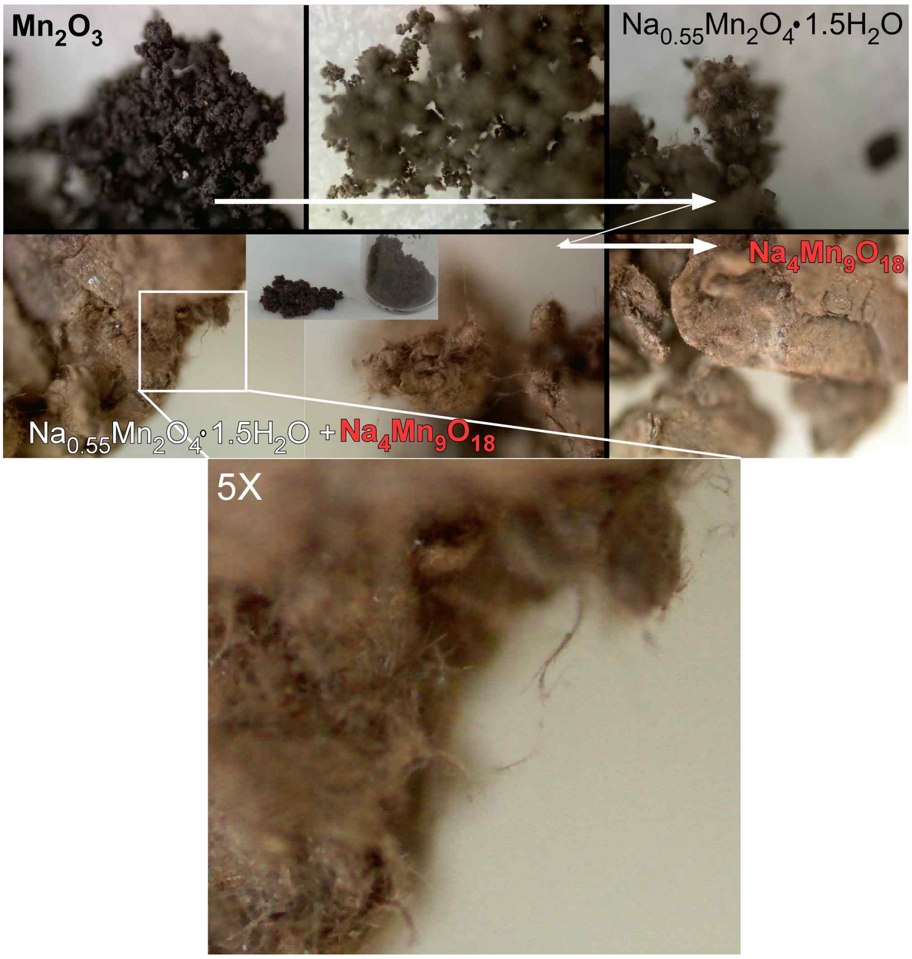

Fig. S11. Optical microscope images of the starting materials and the synthesized samples with reaction time.

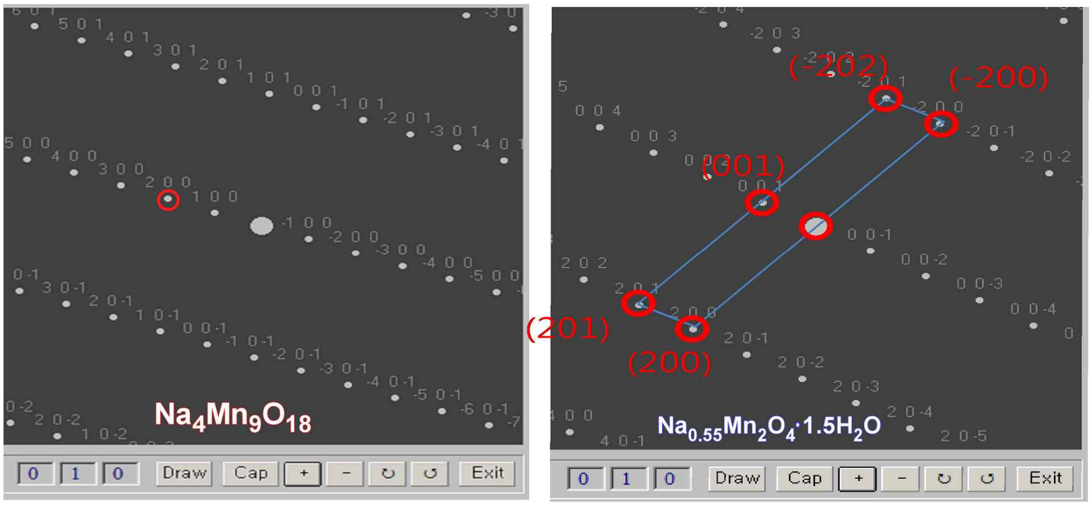

Fig. S12. Simulated electron diffractions patterns for orthorhombic $Na_4Mn_9O_{18}$ (left) and monoclinic $Na_{0.55}Mn_2O_4 \cdot 1.5H_2O$ (right).

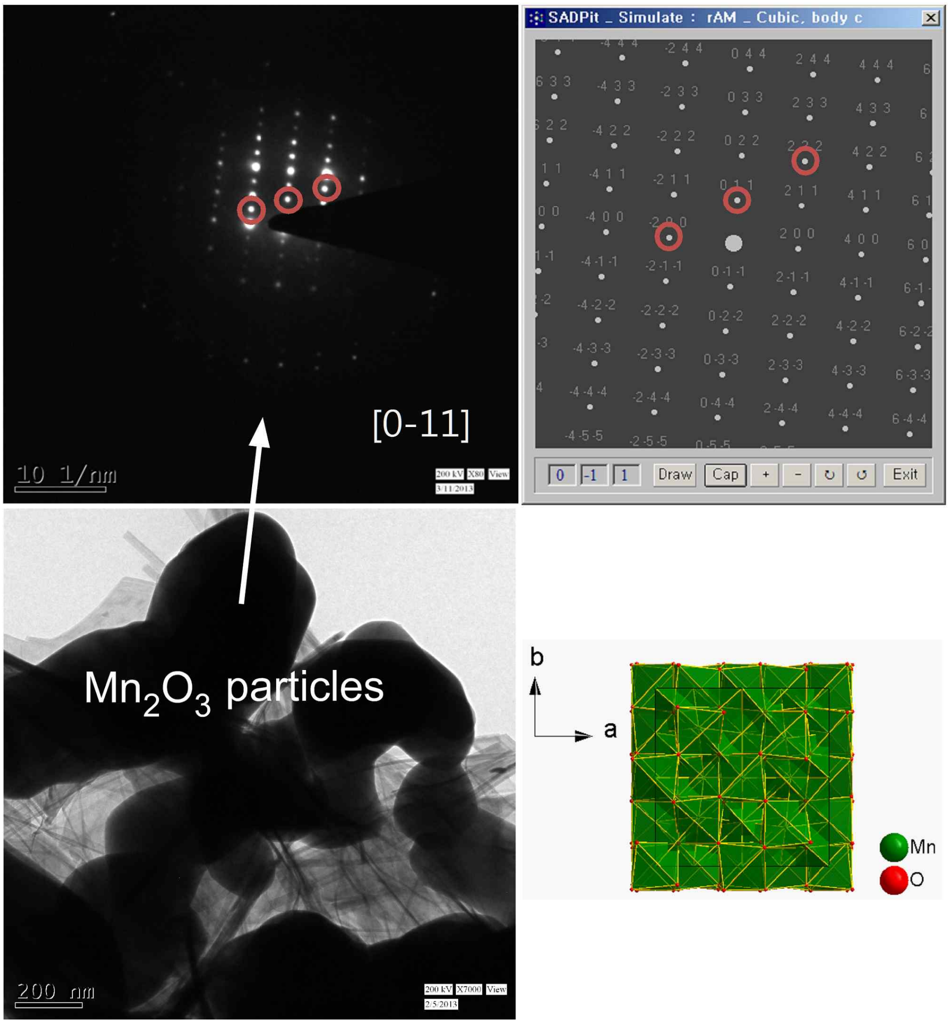

Fig. S13. TEM image (bottom left), SAED (top left), simulated (top right) patterns, and crystal model of unconverted Mn_2O_3 nanoparticles.

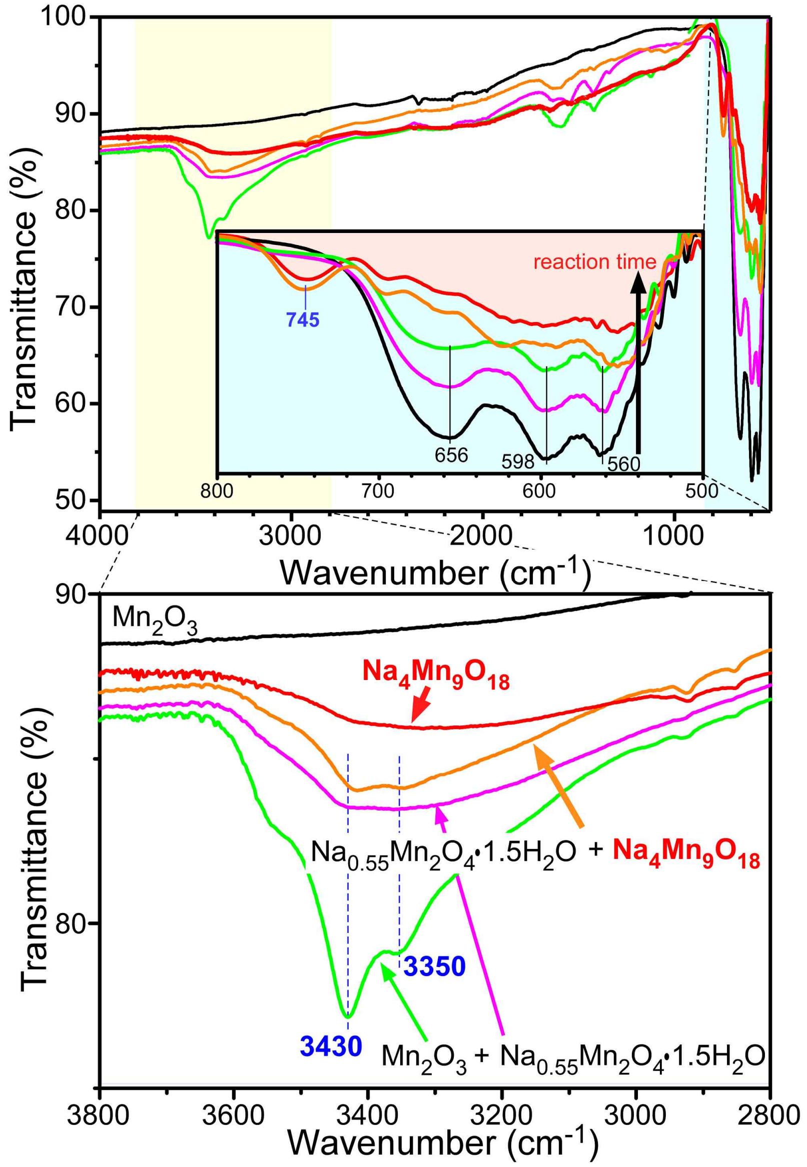

Fig. S14. FT-IR spectra of starting Mn_2O_3 nanoparticles and the synthesized materials with reaction time.

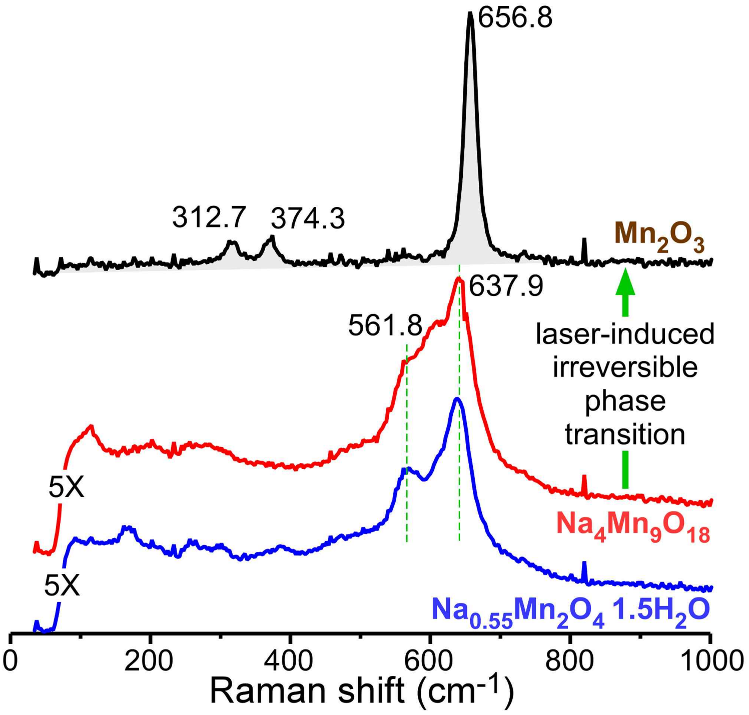

Fig. S15. Raman spectra of $Na_{0.55}Mn_2O_4 \cdot 1.5H_2O$ and $Na_4Mn_9O_{18}$ before and after high power laser exposure (a laser wavelength of 532 nm, a 100×, 0.9NA microscope objective. a laser intensity of 0.19 mW, and 5 sec exposure time).

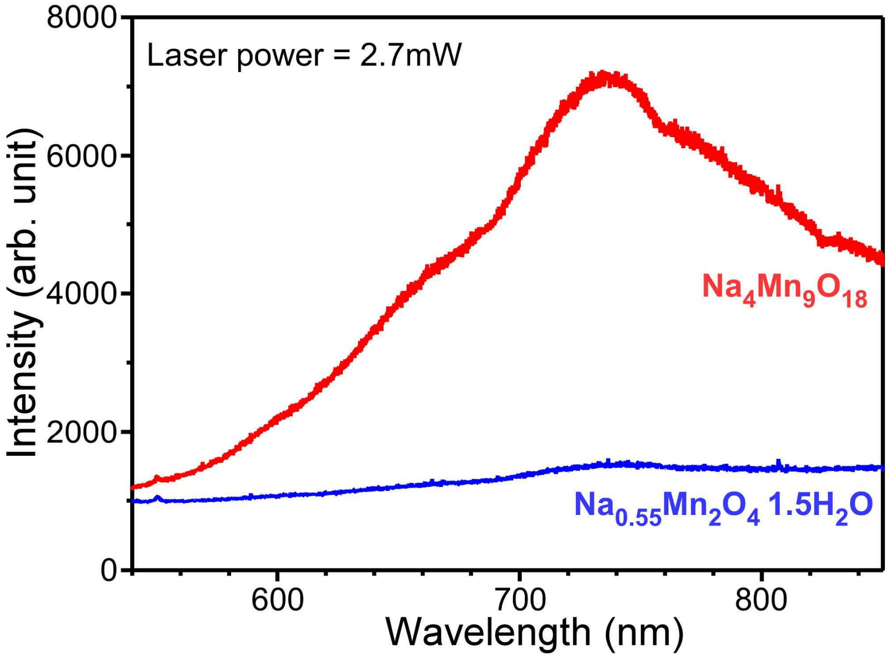

Fig. S16. Photoluminescence spectra of $Na_{0.55}Mn_2O_4 \cdot 1.5H_2O$ and $Na_4Mn_9O_{18}$ taken using a high laser power of 2.7 mW.

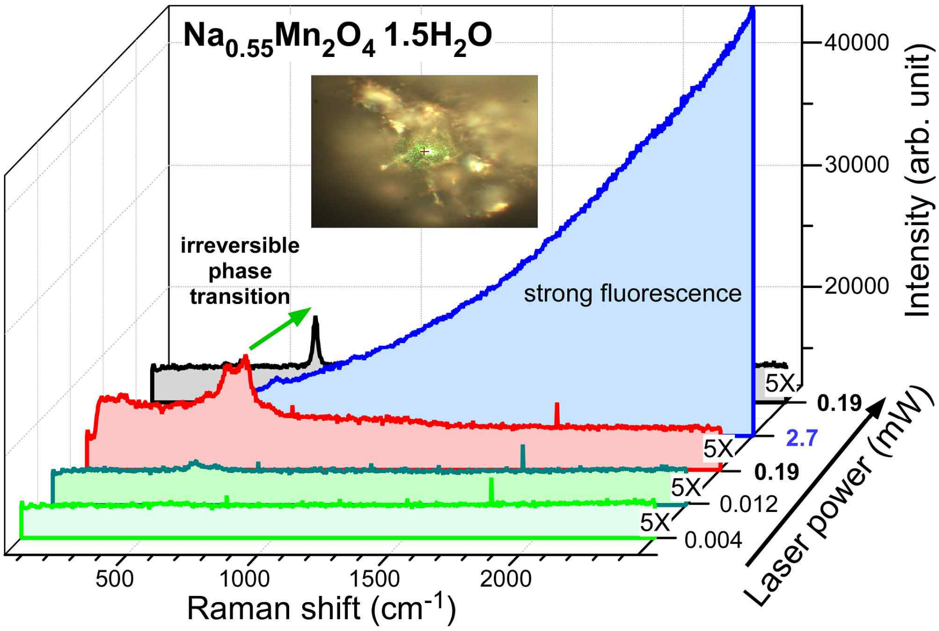

Fig. S17. Raman spectra of $Na_{0.55}Mn_2O_4 \cdot 1.5H_2O$ with laser power (a laser wavelength of 532 nm, a 100×, 0.9NA microscope objective. a laser intensity of 0.19 mW, and 5 sec exposure time)

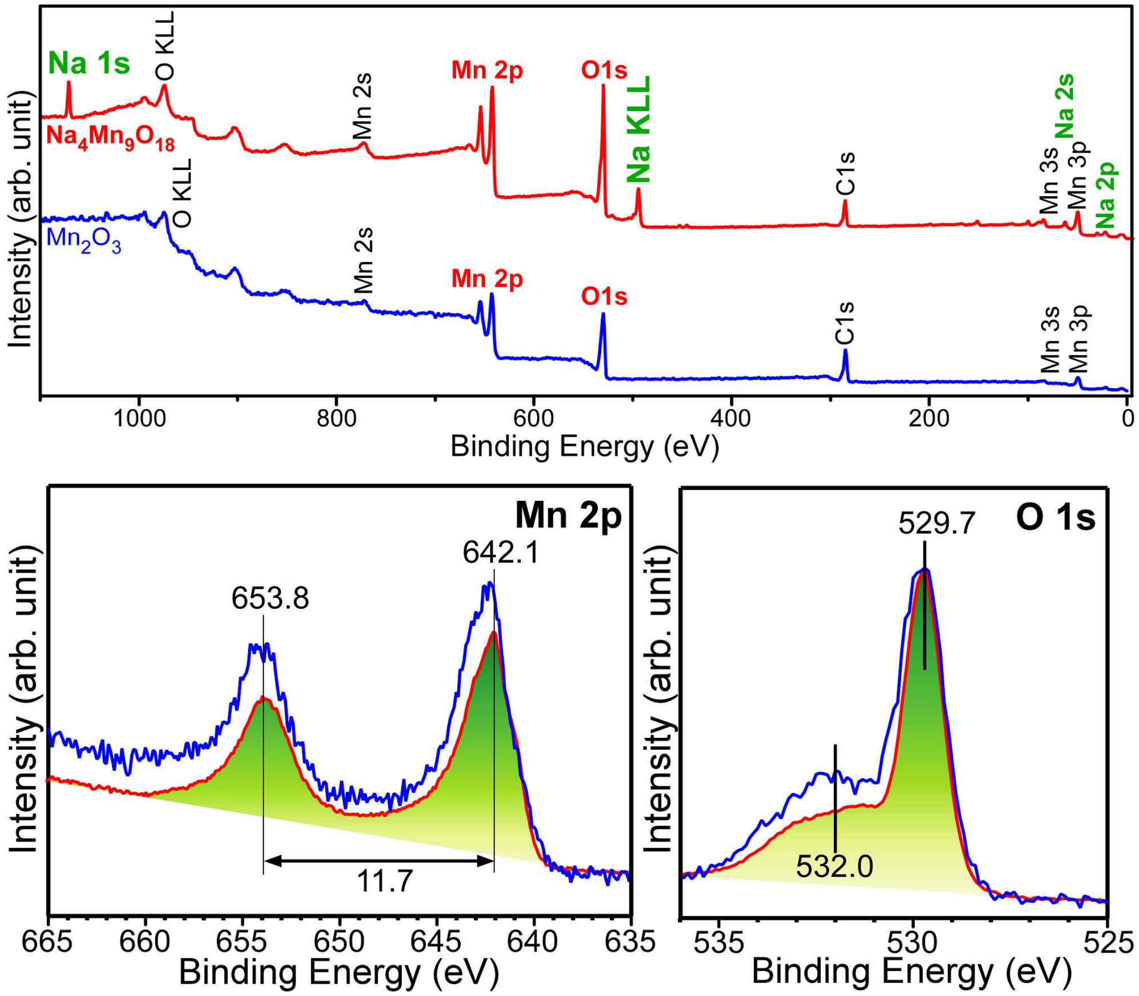

Fig. S18. Survey (top) normalized high resolution Mn 2p and O 1s (bottom) XPS spectra with a common baseline

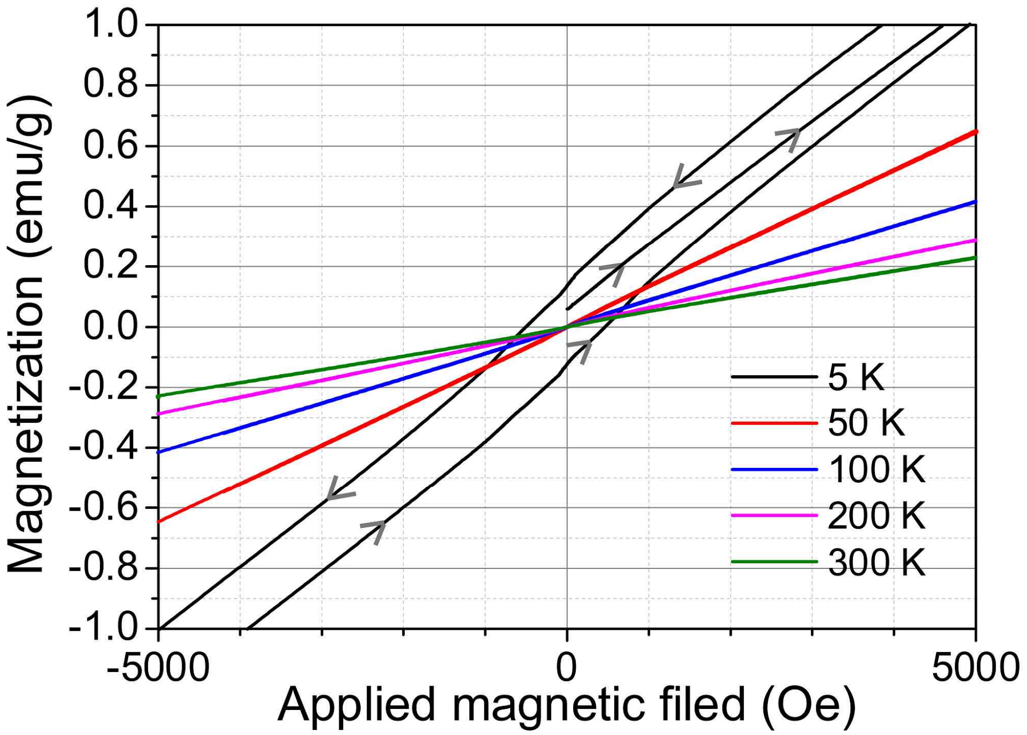

Fig. S19. Magnetization (M-H) curves of $Na_4Mn_9O_{18}$ nanowires measured at various temperatures between -5 kOe and 5kOe.

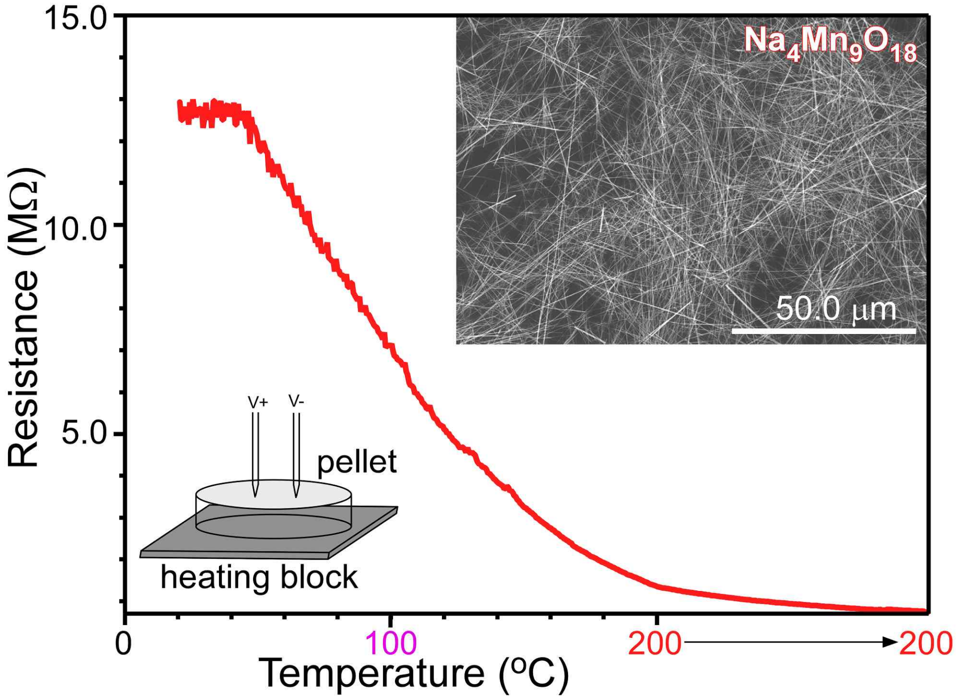

Fig. S20. Surface resistance with sample temperature of $Na_4Mn_9O_8$ nanowires. Inset shows the SEM image of the ultra-long nanowires.

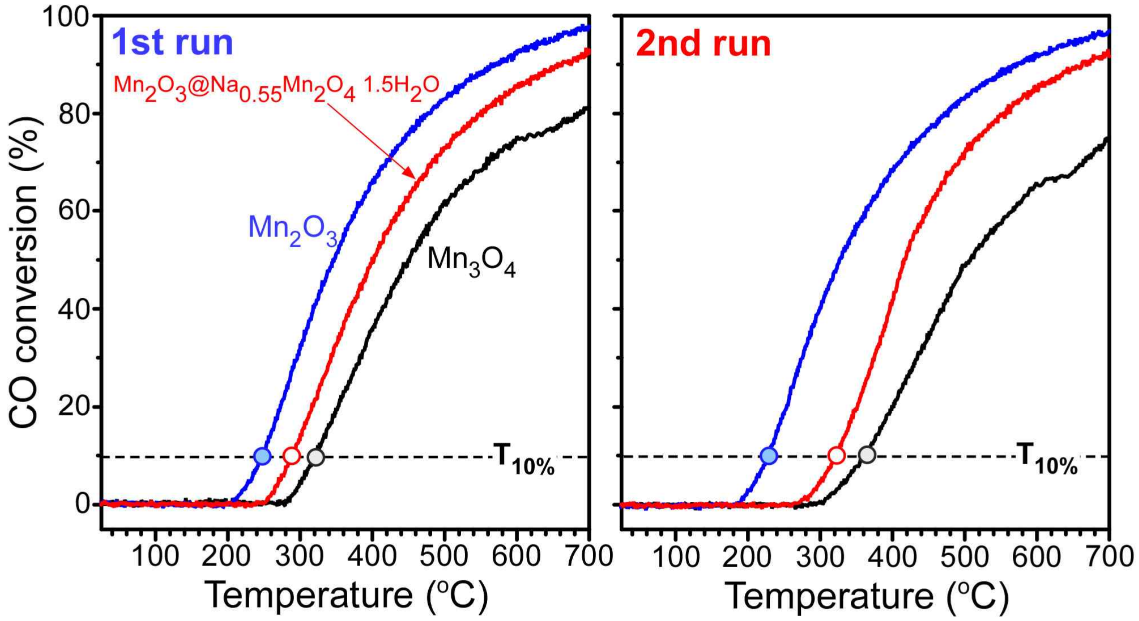

Fig. S21. 1st and 2nd CO oxidation runs of Mn_3O_4, Mn_2O_3 and $Mn_2O_3@Na_{0.55}Mn_2O_4 \cdot 1.5H_2O$ structures.
